# Evaluation of efficacy and safety for compound kushen injection combined with intraperitoneal chemotherapy for patients with malignant ascites: A systematic review and meta-analysis

**DOI:** 10.3389/fphar.2023.1036043

**Published:** 2023-03-03

**Authors:** Hui-Bo Yu, Jia-Qi Hu, Bao-Jin Han, Hui-Juan Cao, Shun-Tai Chen, Xin Chen, Hong-Tai Xiong, Jin Gao, Yan-Yuan Du, Hong-Gang Zheng

**Affiliations:** ^1^ Department of Oncology, Guang’anmen Hospital, China Academy of Chinese Medical Sciences, Beijing, China; ^2^ Graduate School, Beijing University of Chinese Medicine, Beijing, China; ^3^ Centre for Evidence-Based Chinese Medicine, Beijing University of Chinese Medicine, Beijing, China

**Keywords:** compound kushen injection, malignant ascites, intraperitoneal chemotherapy, meta-analysis, botanical drug

## Abstract

**Objectives:** Compound Kushen injection (CKI) combined with intraperitoneal chemotherapy (IPC) is widely used in the treatment of malignant ascites (MA). However, evidence about its efficacy and safety remains limited. This review aimed to evaluate the efficacy and safety of CKI combined with IPC for the treatment of MA.

**Methods:** Protocol of this review was registered in PROSPERO (CRD42022304259). Randomized controlled trials (RCTs) on the efficacy and safety of IPC with CKI for the treatment of patients with MA were searched through 12 electronic databases and 2 clinical trials registration platforms from inception until 20 January 2023. The Cochrane risk-of-bias tool was used to assess the quality of the included trials through the risk of bias assessment. We included RCTs that compared IPC single used or CKI combined with IPC for patients with MA schedule to start IPC. The primary outcome was identified as an objective response rate (ORR), while the secondary outcomes were identified as the quality of life (QoL), survival time, immune functions, and adverse drug reactions (ADRs). The Revman5.4 and Stata17 software were used to calculate the risk ratio (RR) at 95% confidence intervals (CI) for binary outcomes and the mean difference (MD) at 95% CI for continuous outcomes. The certainty of the evidence was assessed according to the GRADE criteria.

**Results:** A total of 17 RCTs were assessed, which included 1200 patients. The risk of bias assessment of the Cochrane risk-of-bias tool revealed that one study was rated high risk and the remaining as unclear or low risk. Meta-analysis revealed that CKI combined with IPC had an advantage in increasing ORR (RR = 1.31, 95% CI 1.20 to 1.43, *p* < 0.00001) and QoL (RR = 1.50, 95% CI 1.23 to 1.83, *p* < 0.0001) when compared with IPC alone. Moreover, the combined treatment group showed a lower incidence of myelosuppression (RR = 0.51, 95%CI 0.40–0.64, *p* < 0.00001), liver dysfunction (RR = 0.33, 95%CI 0.16 to 0.70, *p* = 0.004), renal dysfunction (RR = 0.39, 95%CI 0.17 to 0.89, *p* = 0.02), and fever (RR = 0.51, 95%CI 0.35 to 0.75, *p* = 0.0007) compared to those of the control group. The quality of evidence assessment through GRADE criteria showed that ORR, myelosuppression, and fever were rated moderate, renal dysfunction and liver dysfunction were rated low, and QoL and abdominal pain were rated very low.

**Conclusion:** The efficacy and safety of CKI combined with IPC were superior to that with IPC alone for the treatment of MA, which indicates the potentiality of the treatment. However, more high-quality RCTs are required to validate this conclusion.

**Systematic Review Registration:** [https://www.crd.york.ac.uk/prospero/display_record.php?ID=CRD42022304259], identifier [PROSPERO 2022 CRD42022304259].

## 1 Introduction

Malignant ascites (MA) is a common complication of abdominal malignant tumor (Kalogeraki et al., 2012). The underlying diagnosis is associated with cancer in almost 10% of the patients with ascites, and ascites is the only manifestation in some cancer patients. (Chicago Consensus Working, 2020). MA is common in gynecological malignancies and gastrointestinal malignancies. The most common malignancy is ovarian cancer (37.7%), followed by hepatobiliary and pancreatic cancer (PC) (21%) and gastric cancer (18.3%) (Barni et al., 2011; Chao and Qin, 2019). The aggravation of MA causes distinct abdominal distension, dyspnea (Wang et al., 2017), fatigue, anorexia (Zhong and Zhao, 2015), which reduces the quality of life (QoL) of the patients, shorten the survival time, and signifies a poor prognosis. Although antitumor therapy is developing rapidly, the prevention and treatment of MA are not satisfactory. Some studies have shown that MA is closely associated with shortened survival time. The 1-year survival rate of MA is <10% (Chao and Qin, 2019). An effective control of MA is of great significance for prolonging the survival time and improving the QoL of patients with advanced cancer. Intraperitoneal chemotherapy (IPC) is widely used in the treatment of MA, as it allows increasing the local drug concentration, prolonging the contact time between drugs and cancer cells to kill cancer cells or tiny metastases, and decrease the peritoneal permeability to control MA (He et al., 2009). However, not all patients with MA can benefit from IPC because of the intolerance of adverse events, such as leukopenia, gastrointestinal reactions, renal dysfunction, fever, and pain (Armstrong et al., 2006).

Natural products have been important sources of drug discovery (Ling, 2020). Several anticancer drugs are derived from natural products and approved by the Food and Drug Administration (FDA) in the United States (Yang et al., 2021). More than 50% of new drug approvals from 1946 to 2019 are natural small molecules and their derivatives (Newman and Cragg, 2020). Compound Kushen injection (CKI) is extracted from natural botanical drugs Kushen (*Sophora flavescens* Aiton [Fabaceae]) and Baituling (*Smilax gaudichaudiana* Kunth [Smilacaceae]). CKI was made in accordance with the Ministry of Health Drug Standards (standard number: WS3-B-2752–97) and approved by China Food and Drug Administration (CFDA) (drug approval number: Z14021230) for cancer treatment in 1995. Since 1995, CKI has been widely used in anti-tumor therapy in China (Zhang L. Q, et al., 2012; Chang et al., 2021). Meanwhile a series of CKI clinical studies (NCT04204382, NCT02346318) for patients with cancer have been approved and performed in the United States. These clinical trials have exhibited that CKI combined with chemotherapy offer more advantages in decreasing MA, delaying the progression of cancer, increasing the disease control rate, prolonging the survival time, promoting the QoL, and decreasing toxic and side effects (Li et al., 2016; Huang, 2018; Xia et al., 2018; Xun et al., 2019). CKI may control MA through a series of anti-tumor activities, such as antioxidant activity (Zhang W. J, et al., 2012), promoting apoptosis (Wu et al., 2022), anti-tumor metastasis (Wang K. X et al., 2021), inhibiting angiogenesis (Wang et al., 2019; Han et al., 2020), anti-multidrug resistance of tumor cells, thereby improving immunity function (Zhang et al., 2019; Yang et al., 2020), suppressing the cell cycle, energy metabolism, and DNA repair pathways (Cui et al., 2019). Some studies have demonstrated that CKI combined with IPC has better efficacy than IPC alone, and the incidence of adverse drug reactions (ADRs) is also low. However, the scientific evidence has not been systematically reviewed. The main goal of the present systematic review was to summarize and screen the literature evidence that meets our inclusion criteria to evaluate the efficacy (including the objective response rate [ORR], QoL, survival time, and immune functions) and safety (including ADRs) of CKI combined with IPC when compared with those of IPC alone in the treatment of MA so as to obtain evidence for the treatment of MA.

## 2 Materials and methods

### 2.1 Study design

The protocol of this review has been registered at PROSPERO (CRD42022304259). The report of this systematic review adheres to the Preferred Reporting Items for Systematic Reviews and Meta-Analysis (PRISMA) Checklist (Supplementary File S1).

### 2.2 Preparation of CKI

CKI consists of Kushen (*Sophora flavescens* Aiton [Fabaceae]) and Baituling [*Smilax gaudichaudiana* Kunth (Smilacaceae)] (Zhao et al., 2014), which was supplied by Zhendong Pharmaceutical Co., Ltd. (Shanxi, China). CKI was prepared in accordance with the guidelines of the Ministry of Health Drug Standards (WS3-B-2752–97, Pharmacopoeia commission of the People’s Republic of China, 1997). Briefly, 1400 g of *Sophora flavescens* Aiton [Fabaceae] and 600 g of *S.milax gaudichaudiana* Kunth [Smilacaceae] were crushed with 1% acetic acid solution (solvent) and dipped for 48 h to percolate, followed by the collection of the percolate and condensation under reduced pressure (<75°C) to an appropriate amount. The residue was then decocted with water twice for 1 h each time, followed by filtration and concentration to a predefined amount. The filtrate was then combined with percolation and ethanol to add up to 65% of the volume, and the solution was kept still to allow the settlement of the particles. The solution was then filtered, ethanol was recovered, and the liquid was concentrated under pressure, with the addition of ethanol to make the ethanol content reach 90%. The resultant solution was kept still, filtered, and subjected to the recovery of all ethanol content. Then, 900 mL of the injection water was added to the filtrate along with 4 g of activated carbon, the mixture was boiled for 20 min, cooled, and then filtered. The pH value was adjusted with 20% sodium hydroxide, water was added for injection to 1000 mL, and the solution was filtered, potted, and sterilized to finally obtain the CKI. Chemical identification was performed in accordance with the procedure of thin-layer chromatography, and the alkaloid content in the matrine (C16H24N20) was ensured to be ≥ 18 mg/mL.

### 2.3 Eligibility criteria

#### 2.3.1 Patients

Patients with ascites were confirmed by imaging examination (computed tomography and/or B-ultrasound). Simultaneously, the cancer cells were detected on ascites cytology. Patients without the restrictions on age or sex were included in the trials. The baseline data of patients (such as the sex, age, type of tumor, histological type, and neoplasm staging) in the two groups were compared.

#### 2.3.2 Interventions

The experimental group received CKI combined with IPC. The control group received IPC only.

#### 2.3.3 Outcomes

The primary outcome of the study was the ORR in accordance with the WHO criteria (Miller et al., 1981). The changes in the tumor condition included complete response (CR), partial response (PR), stable disease (SD) and progressive disease (PD). ORR was defined as CR + PR. The secondary outcomes included the QoL as assessed by Karnofsky Performance Status (KPS); survival time assessed by overall survival (OS), progression-free survival (PFS), and disease-free survival (DFS). Immune function was assessed by the level of CD3^+^, CD4^+^, CD8^+^, and NK cells. Moreover, ADRs, including gastrointestinal reactions, myelosuppression, liver dysfunction, renal dysfunction, abdominal pain, and fever, were evaluated based on the incidence rate. We used ORR, QoL, survival time, and immune functions to evaluate the efficacy of the CKI in the treatment of MA as well as applied the ADRs to evaluate the safety of the CKI in the treatment of MA.

#### 2.3.4 Types of studies

All randomized controlled trials (RCTs) published in English or Chinese language were included in the analyses.

#### 2.3.5 Setting of studies

All patients were included in the ward, with clear dates of case collection, at least one complete course of the treatment cycle, and unlimited follow-up time. All data were collected before the beginning of each trial and after the ending of the treatment course.

### 2.4 Exclusion criteria

(1) Articles whose full text cannot be obtained through electronic database or manual retrieval. (2) Reviews, animal experiments, and other unrelated study types; (3) paper with insufficient data that did not allow further analysis; (4) paper including duplicate data; (5) paper lacking a suitable control group; (6) study in which treatment for MA included other anti-tumor drugs, except for chemotherapy drugs and CKI.

### 2.5 Literature searching

All RCTs, both in English or Chinese languages, that were published until 20 January 2023 were searched on the following electronic databases and clinical trials registration platforms: PubMed, EMBASE, the Cochrane Central Register of Controlled Trials (CENTRAL), Turning Research into Practice (TRIP) medical database, Latin American and Caribbean Health Sciences Literature (LILACS), Alt HealthWatch, Web of Science, Google Scholar, China National Knowledge Infrastructure (CNKI), Chinese Scientific Journal Database (VIP database), Wangfang Data Knowledge Service Platform, Chinese Biomedical Literature Database (CBM), clinicaltrials.gov, and Chinese Clinical Trial Registry.

The searched terms of retrieval strategy were different based on a different database. The following terms were used in the English databases: “Kushen”, “matrine”, “sophora”, “Yanshu”, “CKI”, “ascitic fluid”, “ascites”, “peritoneal fluid”, “peritoneal effusion”, and “random”. The equivalent search words were used in the Chinese databases (the detailed search strategy is illustrated in Supplementary File S2). We searched for additional studies by reviewing the reference lists of the related studies. All studies were searched by two reviewers (HBY and BJH). Any disagreement was sorted through discussion with a third reviewer (JQH).

### 2.6 Study screening and data extraction

Two reviewers (HBY and BJH) independently imported the studies into the Endnote X9 software. After the exclusion of duplicate studies, the remaining studies were independently assessed for eligibility by two reviewers (STC and XC). Any disagreement was resolved through discussion with the third reviewer (HJC). Two reviewers (HTX and JG) imported the relevant data into EpiData 3.1. The extracted information included the following: 1. General information (title, first author, year(s), etc.); 2. Methodological information (i.e., study design type, random scheme concealment, random allocation method, randomization blind method, statistical analyst blinded, the loss of follow-up, baseline comparability, and selective reporting); 3. Participants information (i.e., diagnostic criteria, inclusion criteria, source, sample size, age, gender, and the types of carcinoma); 4. Intervention information (i.e., chemotherapy regimens, the dose of drugs, and treatment duration); 5. Study outcomes.

### 2.7 Methodological quality assessment

Two reviewers (STC and YYD) evaluated the enrolled studies independently, and any disagreements arising from this process were resolved through discussion with a third reviewer (JQH). Any risk of bias was assessed with the use of the Cochrane risk-of-bias tool to ensure the quality of included studies, in which the following seven domains were assessed: Random sequence generation, allocation concealment, blinding of participants and personnel, blinding of outcome assessment, incomplete outcome data, selective reporting, and other bias. An overall judgment for each domain included three response options (low/unclear/high risk of bias) (Julian et al., 2022).

### 2.8 Data synthesis

Two reviewers (HBY and BJH) conducted a meta-analysis on the included studies using RevMan5.4 and Stata17 software. The data was summarized by using risk ratio (RR) calculations and 95% confidence intervals (CI) for binary outcomes or mean difference (MD) with 95% CI for continuous outcomes.

Statistical heterogeneity among all trials were evaluated by the *I* ^*2*^ test. Meta-analysis was conducted in case of no significant clinical and statistical heterogeneity (*I* ^*2*^ < 75%) among the included trials. If *I* ^*2*^ ≤ 25%, a fixed-effect model (FEM) was used to pool the data. If 25% < *I* ^*2*^ < 75%, we first assessed the sources of the heterogeneity. If the statistical heterogeneity was explained successfully by sensitive analysis or subgroup analysis (*I* ^*2*^ ≤ 25%), the FEM was used to pool the data; otherwise, the random-effects model (REM) was used in the meta-analysis. Pooling analysis was not performed in the case of a significant statistical heterogeneity (*I* ^*2*^ ≥ 75%) among the trials, as subgroup analysis could not explain the huge heterogeneity. The funnel plot and Begg’s test were performed to explore the potential publication bias in case of ≥10 trials in the meta-analysis.

### 2.9 Additional analysis

Subgroup analyses were conducted to determine the effects of the chemotherapy regimen, the dose of CKI, course of treatment, cancer types, and KPS score. Sensitivity analysis was performed to challenge the robustness of the primary analysis for trials with/without a high risk of bias and for meta-analyses conducted using the FEM or the REM.

### 2.10 Assessment of evidence quality

Two reviewers (HBY and JQH) independently evaluated the quality of the evidence for outcomes with meta-analysis with reference to the Grading of Recommendations Assessment Development and Evaluation criteria (GRADE), which including the following five domains: risk of bias, inconsistency of trials, indirectness evidence, imprecision of results, and publication bias. The evidence was assessed on four different levels: high, moderate, low, or very low. Any disagreements were resolved through consensus or discussion with a third reviewer (HGZ).

## 3 Results

### 3.1 Inclusion of studies

**FIGURE 1:**
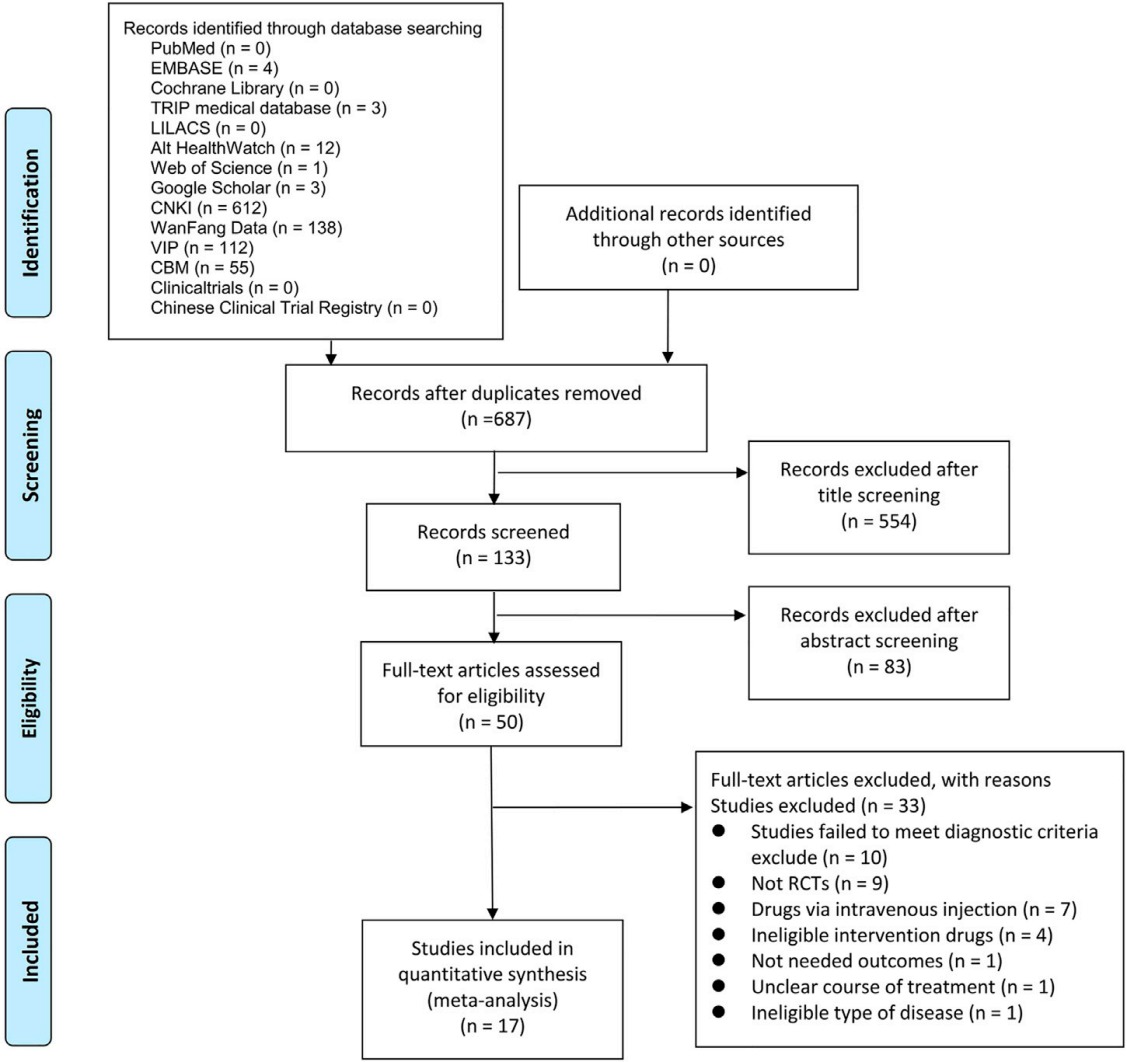
Study flow chart.

A total of 940 potential studies were assessed, from which only 687 articles were included after removing the duplicated articles. A total of 554 articles were excluded through the title screening, and 133 articles remained after the abstracts screening. A total of 50 articles were subjected to full-text screening. Base on the exclusion criteria, 33 studies were excluded. Finally, 17 eligible studies were included for data extraction and quantitative synthesis. The specific study process is depicted in Figure 1.

### 3.2 Characteristics of included studies

A total of 17 RCTs, comprising 1,200 patients, were presented in this review. The sample size of the studies ranged from 32 to 106. In one study (Sun and Yang, 2009), the age of the patients was not reported, and, in another (Li et al., 2016), the sex of the patients was not reported. The age of the patients in the other study was between 28 and 80 years. A total of 13 studies involved gastric cancer and nine studies involved ovarian cancer, which was the most common causes of MA. In all studies, MA was controlled by CKI combined with IPC. Among the included studies, 11 studies (Cheng et al., 2006; Sun and Yang, 2009; Huang, 2010; Chen and Chen, 2011; Zhang Y et al., 2012; Jia et al., 2015; Zhang et al., 2016; Tao et al., 2017; Jiang et al., 2018; Wu, 2018; Zhang Y et al., 2020) used cisplatin, 2 studies (Zhang D, et al., 2017; Gao L et al., 2018) used lobaplatin, 2 studies (Tang et al., 2015; Li et al., 2016) used paclitaxel liposome, 1 study used carboplatin, and 1 study (Ma, 2016) used fluorouracil. The dosage of CKI was 20–40 mL. A total of 14 studies (Cheng et al., 2006; Huang, 2010; Chen and Chen, 2011; Zhang L. Q, et al., 2012; Tang et al., 2015; Li et al., 2016; Ma, 2016; Zhang et al., 2016; Tao et al., 2017; Zhang T et al., 2017; Gao S. T et al., 2018; Wu, 2018; Zhang D et al., 2020; Zhou, 2022) reported the ORR and seven studies (Cheng et al., 2006; Huang, 2010; Chen and Chen, 2011; Jia et al., 2015; Li et al., 2016; Zhang et al., 2016; Zhou, 2022) reported the change in the QoL of the patients. Adverse events were reported in 12 studies (Sun and Yang, 2009; Huang, 2010; Chen and Chen, 2011; Jia et al., 2015; Tang et al., 2015; Li et al., 2016; Zhang et al., 2016; Tao et al., 2017; Zhang D, et al., 2017; Gao L et al., 2018; Jiang et al., 2018; Zhang Y et al., 2020), 11 studies (Sun and Yang, 2009; Huang, 2010; Chen and Chen, 2011; Tang et al., 2015; Li et al., 2016; Zhang et al., 2016; Tao et al., 2017; Zhang T et al., 2017; Gao S. T et al., 2018; Jiang et al., 2018; Zhang Y et al., 2020) reported gastrointestinal reactions, 11 studies (Sun and Yang, 2009; Huang, 2010; Chen and Chen, 2011; Tang et al., 2015; Li et al., 2016; Zhang et al., 2016; Tao et al., 2017; Zhang D, et al., 2017; Gao L et al., 2018; Jiang et al., 2018; Zhang Y et al., 2020) reported myelosuppression, 3 studies (Jia et al., 2015; Tao et al., 2017; Gao S. T et al., 2018) reported liver dysfunction, 3 studies (Jia et al., 2015; Tao et al., 2017; Gao L et al., 2018) reported renal dysfunction, 2 studies (Sun and Yang, 2009; Huang, 2010) reported abdominal pain, and 7 studies (Huang, 2010; Jia et al., 2015; Tang et al., 2015; Li et al., 2016; Zhang et al., 2016; Jiang et al., 2018; Zhang Y et al., 2020) reported fever. The basic characteristics of the included studies are shown in Table 1.

**TABLE 1:** Main characteristics of studies included in the meta-analysis.

Study ID	Country	Simple size (T/C)	Male/Female	Age (mean or mean ± SD)	KPS score	Cancer type	Intervention	Control	Course of treatment	Outcome
Chen et al. (2011)	China	57 (31/26)	36/21	53.1 ± 15.8	40–70	Gastric cancer, lung cancer, ovarian cancer, breast cancer, liver cancer	CKI 30 mL + Cisplatin 40 mg	Cisplatin 60 mg	1/w, 4w	1234
Cheng et al., 2006	China	62 (34/28)	34/28	31–80	<65	Gastric cancer, liver cancer, ovarian cancer, colon cancer, peritoneal mesothelioma, pancreatic cancer, others	CKI 40 mL + Cisplatin 40 mg	Cisplatin 40 mg	1/w, 4w	12
Gao S. T et al. (2018)	China	98 (49/49)	51/47	28–65, Median age:46	≥60	Gastric cancer, ovarian cancer, colon cancer, uterine cervix cancer	CKI 40 mL + Lobaplatin 40mg/m2	Lobaplatin 40mg/m2	1/w, 3w	13456
Huang C 2010	China	90 (45/45)	48/42	65	≥50	Colorectal cancer, liver cancer, gastric cancer, ovarian cancer, breast cancer, pancreatic cancer, malignant lymphoma, gallbladder cancer	CKI 40 mL + Cisplatin 40 mg	Cisplatin 40 mg	1/w, 4w	123478
Jia et al. (2015)	China	42 (22/20)	20/22	T:37–68, Median age:55	>60	Gastric cancer, ovarian cancer, colon cancer	CKI 30 mL + Cisplatin 80 mg	Cisplatin 80 mg	1/w, 4w	2568
				C:39–73, Median age:57						
Jiang et al. (2018)	China	100 (50/50)	56/44	T:54.10 ± 6.77	≥60	Gastric cancer, ovarian cancer, colon cancer	CKI 30 mL + Cisplatin 100 mg	Cisplatin 100 mg	1/w, 4w	3489
				C:54.23 ± 6.80						
Li et al. (2016)	China	65 (33/32)	unclear	T:54.2 ± 4.3	≥80	Gastric cancer	CKI 30 mL + Paclitaxel Liposome 60 mg	Paclitaxel Liposome 60 mg	1/w, 8w	12348
				C:53.6 ± 4.7						
Ma (2016)	China	100 (50/50)	77/23	T:54.34 ± 2.24	unclear	Liver cancer	CKI 30 mL + 1000 mg/m2 5-Fu	1000 mg/m2 5-Fu	1/w, 2-4w	1
				C:54.31 ± 2.47						
Sun and Yang (2009)	China	56 (29/27)	0/56	—	unclear	Ovarian cancer	CKI 20 mL + Cisplatin 60 mg	Cisplatin 60 mg	1/w, 3w	347
Tang et al. (2015)	China	32 (16/16)	15/17	T:49.8 ± 5.7	unclear	Gastric cancer, liver cancer, ovarian cancer, pancreatic cancer, colorectal cancer	CKI 30 mL + Paclitaxel Liposome 60 mg	Paclitaxel Liposome 60 mg	2/21d, 21d/cycle, 3cycles	1348
				C:54.6 ± 6.1						
Tao et al. (2017)	China	86 (43/43)	47/39	T:63.05 ± 6.32	<60	Gastric cancer, liver cancer, ovarian cancer, pancreatic cancer, colorectal cancer	CKI 30 mL + Cisplatin 100 mg	Cisplatin 100 mg	1/w, 3w	13456
				C:65.37 ± 6.15						
Wu (2018)	China	106 (53/53)	74/32	T:31–72	unclear	Liver cancer	CKI 30 mL + Cisplatin 16 mg/m2	Cisplatin 16 mg/m2	1/w, 2-4w	1
				C:35–76						
Zhang et al. (2016)	China	96 (48/48)	51/45	T:56.33 ± 8.24	≥60	Gastric cancer	CKI 30 mL + Cisplatin 80 mg	Cisplatin 80 mg	1/w, 4w	123489
				C:56.57 ± 8.63						
Zhang L. Q, et al. (2012)	China	48 (27/21)	28/20	43–72, Median age:54	≥50	Gastric cancer, liver cancer, colorectal cancer, mesenchymoma, gallbladder cancer	CKI 30 mL + Cisplatin 40 mg/m2	Cisplatin 40 mg/m2	1/w, 4w	19
Zhang D et al. (2017)	China	40 (20/20)	0/40	54.4 ± 10.3	≥60	Ovarian cancer	CKI 40 mL + Lobaplatin 30mg/m2	Lobaplatin 30mg/m2	1/w, 2-4w	134
Zhang Y et al. (2020)	China	74 (37/37)	40/34	T:56.06 ± 5.09	≥60	Gastric cancer, liver cancer, colon cancer, pancreatic cancer	CKI 30 mL + Cisplatin 80 mg	Cisplatin 80 mg	1/w, 3w	13489
				C:55.89 ± 5.12						
Zhou 2022	China	48 (24/24)	28/20	T:69.7 ± 6.52	≥60	Lung cancer, esophagus cancer, breast cancer, gastric cancer, liver cancer, colon cancer, lymphoma	CKI 30 mL + Carboplatin 40 mg	Carboplatin 40 mg	1/w, 1-4w	12
				C:69.2 ± 7.03						

SD, standard deviation; KPS, Karnofsky performance status; N, number of patients; T/C, treatment group (CKI, combined with IPC)/control group (IPC, alone). 1. Objective response rate; 2. KPS, score; 3. Gastrointestinal reactions; 4. Myelosuppression; 5. Liver damage; 6. Renal damage; 7. Abdominal pain; 8. Fever; 9. Immune function.

### 3.3 Methodological quality

**FIGURE 2:**
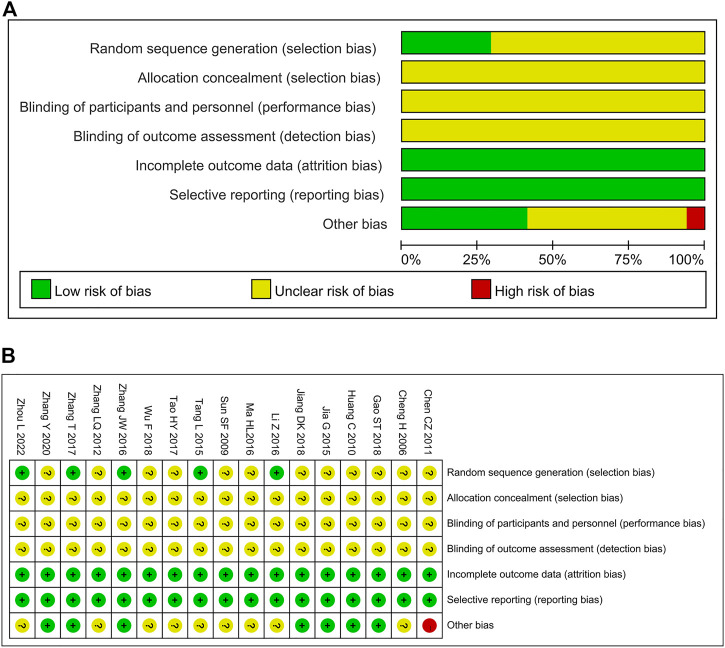
Risk of bias of included studies. **(A)** Risk of bias graph; **(B)** Risk of bias summary.

We assessed the risk of bias for 17 included studies. For “Random sequence generation,” five studies (Tang et al., 2015; Li et al., 2016; Zhang et al., 2016; Zhang T et al., 2017; Zhou, 2022) were assessed as low risk base on the random number table method. Other studies (Cheng et al., 2006; Sun and Yang, 2009; Huang, 2010; Chen and Chen, 2011; Zhang W. J, et al., 2012; Jia et al., 2015; Ma, 2016; Tao et al., 2017; Gao L et al., 2018; Jiang et al., 2018; Wu, 2018; Zhang D et al., 2020) only mentioned “random” and did not describe the specific methods, and hence they were assessed as unclear. None of studies reported allocation concealment and blind methods; therefore, the risk was assessed as unclear. For “Incomplete outcome data,” all the included studies were evaluated as low risk because of the potential completeness of the data. For “Selective reporting,” all studies were assessed as low risk because their experimental analysis methods were consistent with the preset methods. For “Other bias,” one study (Chen and Chen, 2011) was assessed as high risk because the dose of chemotherapy drugs was inconsistent between the experimental and the control groups. Due to the presence of unclear/insufficient information related to age, KPS score, sex, and CKI manufacturers in 9 trials (Cheng et al., 2006; Sun and Yang, 2009; Zhang L. Q, et al., 2012; Tang et al., 2015; Li et al., 2016; Ma, 2016; Tao et al., 2017; Wu, 2018; Zhou, 2022) were assessed as unclear. A summary of the results of risk of bias assessment for the included studies is shown in Figure 2.

### 3.4 Primary outcome

#### 3.4.1 Objective response rate

**FIGURE 3:**
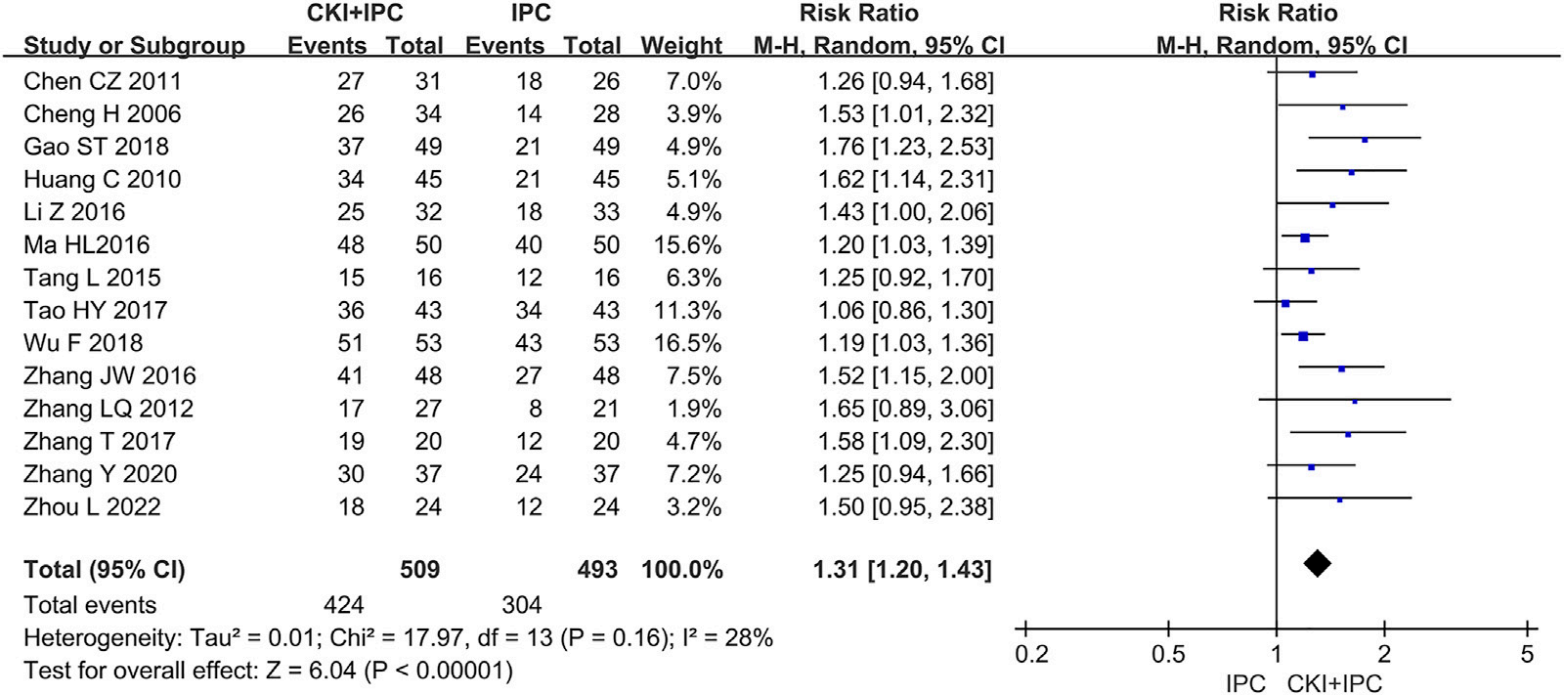
Forest plot and pooled risk ratios for the association of objective response rate (ORR) with CKI + IPC and IPC. CKI, Compound Kushen Injection; IPC, intraperitoneal chemotherapy.

A total of 1002 patients in 14 studies (Chen and Chen, 2011; Cheng et al., 2006; Gao S. T et al., 2018; Huang, 2010; Li et al., 2016; Ma, 2016; Tang et al., 2015; Tao et al., 2017; Wu, 2018; Zhang et al., 2016; Zhang W. J, et al., 2012; Zhang D, et al., 2017; Zhang D et al., 2020+; Zhou, 2022) reported ORR. Meta-analysis showed that, when compared with IPC alone, CKI combined with IPC had an advantage in increasing ORR (REM, RR = 1.31, 95%CI 1.20 to 1.43, 1002 participants, *I* ^*2*^ = 28%, *p* < 0.00001) (Shown in Figure 3).

#### 3.4.2 Subgroup analysis of ORR

**FIGURE 4:**
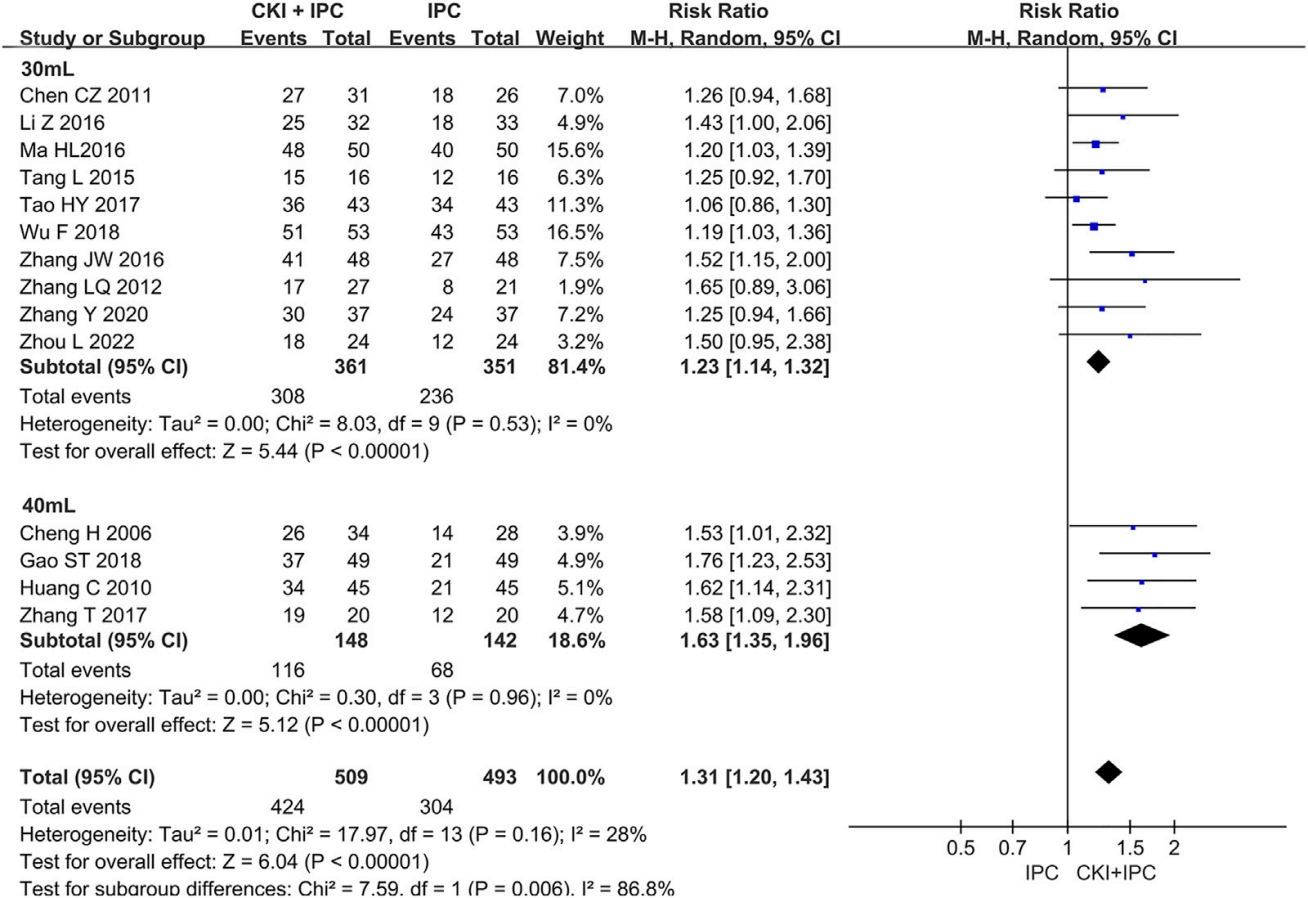
Forest plot and pooled risk ratios for the association of objective response rate (ORR) with CKI + IPC and IPC. Subgroup analysis of different CKI dosage.

**FIGURE 5:**
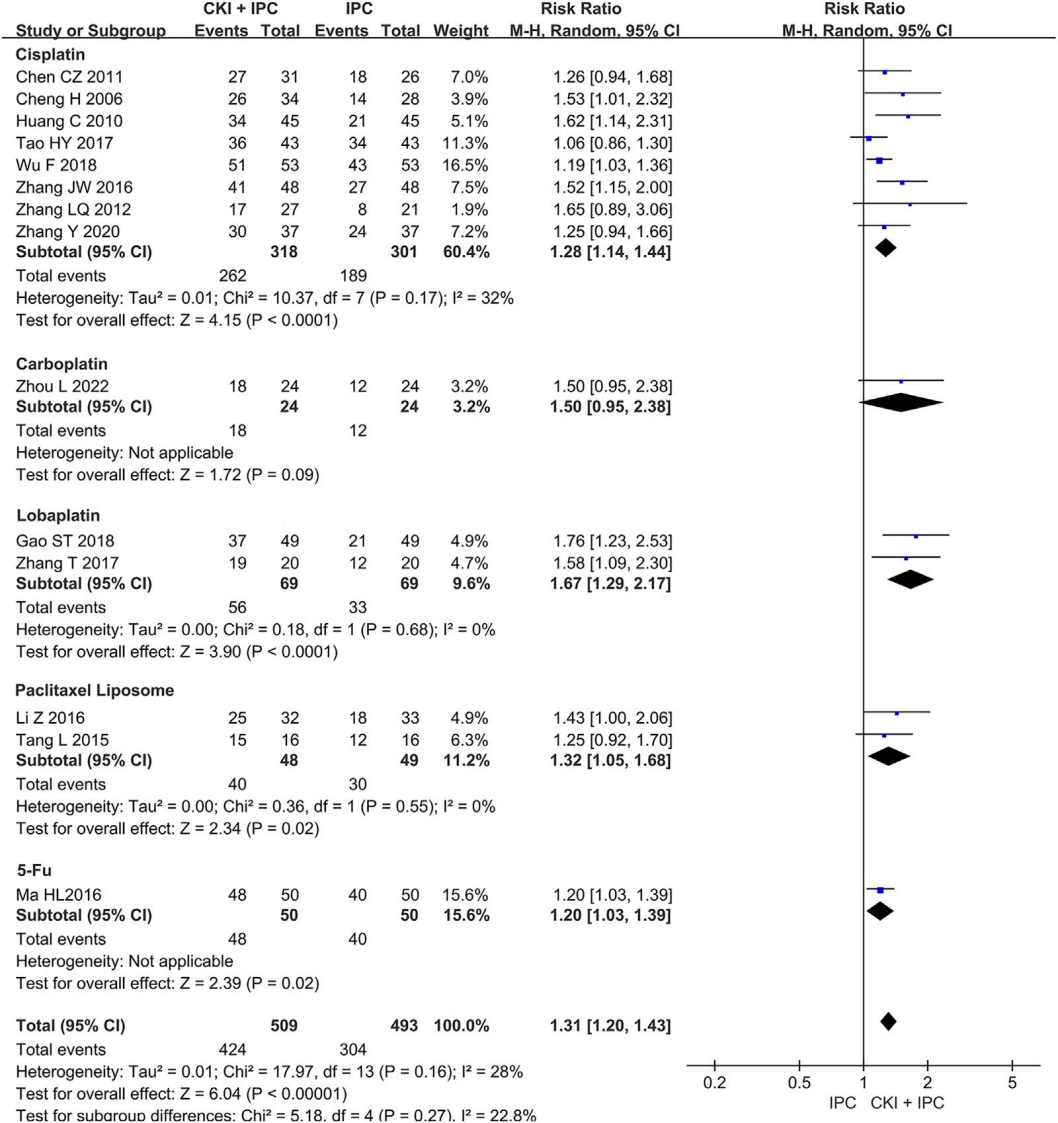
Forest plot and pooled risk ratios for the association of objective response rate (ORR) with CKI + IPC and IPC. Subgroup analysis of different chemotherapy regimen.

Subgroup analysis of ORR was performed base on the chemotherapy regimen, dose of CKI, the course of treatment, cancer types, and KPS score (Shown in Table 2). The dose of CKI was 30 mL and 40 mL. Subgroup analysis revealed that higher dosage of CKI might have a better effect on ORR (Shown in Figure 4). The chemotherapy regimen included cisplatin, lobaplatin, carboplatin, paclitaxel liposome, and 5-Fu. Subgroup analysis revealed that CKI may have more advantage when combined with lobaplatin (Shown in Figure 5). Subgroup analysis classification by the course of treatment, cancer types, and KPS score did not explain the heterogeneity (Shown in Supplementary Figure S1–S3). Furthermore, subgroup analysis on the cancer types showed that the ovarian cancer subgroup had the highest effect value (REM, RR = 1.58, 95%CI 1.09 to 2.30, 31 participants, *p* = 0.02), followed by gastric cancer (REM, RR = 1.49, 95%CI 1.19 to 1.85, 111 participants, *p* = 0.0004), mixed cancer (REM, RR = 1.34, 95%CI 1.17 to 1.53, 404 participants, *p* < 0.0001), and liver cancer (REM, RR = 1.19, 95%CI 1.08 to 1.32, 182 participants, *p* = 0.0007). However, the number of studies reported by a single cancer subgroup was very small to draw convincing conclusions. Further, we used meta -regression to determine the degree of correlation between subgroups and intervention effects. The result indicated that only CKI dose had statistically significant (*p* = 0.042) (shown in Table 2).

**TABLE 2:** Subgroup analysis of the ORR.

Subgroup	Number of trials	RR,95%CI	Z	*P*	Heterogeneity		Meta-regression
					*I* ^*2*^	*P*_*h*_	*p*
**Subgroups analysis according to chemotherapy regimen**							0.766
Cisplatin	8	1.28 [1.14, 1.44]	4.15	<0.0001	32%	0.17	
Carboplatin	1	1.50 [0.95, 2.38]	1.72	0.09	Not applicable	Not applicable	
Lobaplatin	2	1.67 [1.29, 2.17]	3.90	<0.0001	0%	0.68	
Paclitaxel Liposome	2	1.32 [1.05, 1.68]	2.34	0.02	0%	0.55	
5-Fu	1	1.20 [1.03, 1.39]	2.39	0.02	Not applicable	Not applicable	
**Subgroups analysis according to CKI dosage**							0.042
30 mL	10	1.23 [1.14, 1.32]	5.44	<0.00001	0%	0.53	
40 mL	4	1.63 [1.35, 1.96]	5.12	<0.00001	0%	0.96	
**Subgroups analysis according to course of treatment**							0.206
<4w	7	1.26 [1.12, 1.41]	3.08	0.0001	41%	0.12	
≥4w	7	1.42 [1.25, 1.62]	5.32	<0.00001	0%	0.85	
**Subgroups analysis according to cancer types**							0.860
Gastric cancer	2	1.49 [1.19, 1.85]	3.54	0.0004	0%	0.80	
Liver cancer	2	1.19 [1.08, 1.32]	3.37	0.0007	0%	0.91	
Ovarian cancer	1	1.58 [1.09, 2.30]	2.42	0.02	Not applicable	Not applicable	
Unable to classification	9	1.34 [1.17, 1.53]	4.32	<0.0001	29%	0.19	
**Subgroups analysis according to KPS score**							0.341
≥60	7	1.38 [1.18, 1.61]	4.04	<0.0001	42%	0.11	
<60	4	1.44 [1.20, 1.74]	3.82	0.0001	0%	0.65	
Unclear	3	1.20 [1.09, 1.32]	3.64	0.0003	0%	0.95	

### 3.5 Secondary outcomes

#### 3.5.1 Survival time

No study reported this outcome.

#### 3.5.2 Quality of life

**FIGURE 6:**
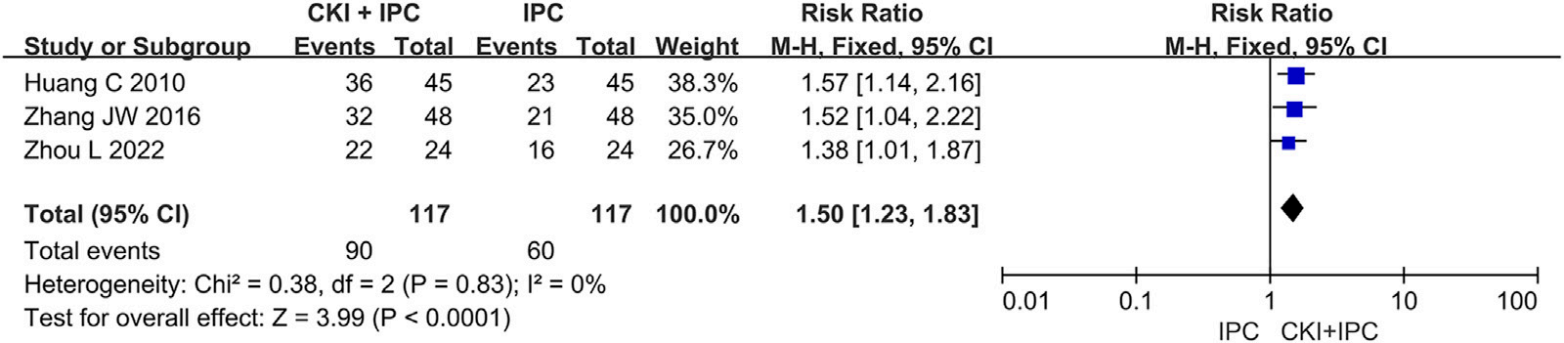
Forest plot and pooled risk ratios for the association of QoL (continuous data) with CKI + IPC and IPC.

A total of 234 patients in 3 studies (Huang, 2010; Zhang et al., 2016; Zhou, 2022) reported the QoL by dichotomous data. Meta-analysis revealed that CKI combined with IPC could increase number of patients whose added KPS scores >10 when compared with IPC alone based on the baseline level (FEM, RR = 1.50, 95%CI 1.23 to 1.83, 234 participants, *I* ^*2*^ = 0%, *p* < 0.0001). (Shown in Figure 6).

Meanwhile, 4 studies (Cheng et al., 2006; Chen and Chen, 2011; Jia et al., 2015; Li et al., 2016) with 226 patients were reported by continuous data based on the KPS scale. Owing to the huge heterogeneity, we could not conduct a meta-analysis. However, subgroup analysis showed that the dosage of CKI could be a reason for heterogeneity; CKI (30 mL) plus IPC might have a better effect on improving QoL when compared to IPC alone. (REM, RR = 11.05, 95%CI 9.54 to 12.56, 164 participants, *I* ^*2*^ = 0%, *p* < 0.00001) (Shown in Supplementary Figure S4).

#### 3.5.3 Immune functions

A total of 4 studies (Zhang L. Q, et al., 2012; Zhang et al., 2016; Jiang et al., 2018; Zhang D et al., 2020) reported immune functions including the level of CD3^+^, CD4^+^, CD8^+^, CD4^+^/CD8^+^. Owing to the huge heterogeneity, we could not conduct a meta-analysis. All studies showed that the combined group could increase the level of CD3^+^, CD4^+^, CD4^+^/CD8^+^. Furthermore, three studies reported that the combined group could decrease the level of CD8^+^, and another study reported the opposite results. Only one study (Zhang et al., 2016) reported the level of NK cells, which suggested that the combined group could increase the level of NK cells. The specific data is depicted in Supplementary Table S1.

#### 3.5.4 ADRs

A total of 12 studies (Sun and Yang, 2009; Huang, 2010; Chen and Chen, 2011; Jia et al., 2015; Tang et al., 2015; Li et al., 2016; Zhang et al., 2016; Tao et al., 2017; Zhang T et al., 2017; Gao L et al., 2018; Jiang et al., 2018; Zhang Y et al., 2020) reported ADRs in total. A total of 11 studies (Sun and Yang, 2009; Huang, 2010; Chen and Chen, 2011; Tang et al., 2015; Li et al., 2016; Zhang et al., 2016; Tao et al., 2017; Zhang D, et al., 2017; Gao S. T et al., 2018; Jiang et al., 2018; Zhang D et al., 2020) with 794 patients reported gastrointestinal reactions (nausea or/and vomiting or/and diarrhea), 11 studies (Sun and Yang, 2009; Huang, 2010; Chen and Chen, 2011; Tang et al., 2015; Li et al., 2016; Zhang et al., 2016; Tao et al., 2017; Zhang T et al., 2017; Gao L et al., 2018; Jiang et al., 2018; Zhang Y et al., 2020) with 794 patients reported myelosuppression, three studies (Jia et al., 2015; Tao et al., 2017; Gao S. T et al., 2018) with 226 patients reported liver dysfunction, three studies (Jia et al., 2015; Tao et al., 2017; Gao L et al., 2018) with 226 patients reported renal dysfunction, two studies (Sun and Yang, 2009; Huang, 2010) with 146 patients reported abdominal pain, and seven studies (Huang, 2010; Jia et al., 2015; Tang et al., 2015; Li et al., 2016; Zhang et al., 2016; Jiang et al., 2018; Zhang D et al., 2020) with 499 patients reported fever (Shown in Table 1).

**FIGURE 7:**
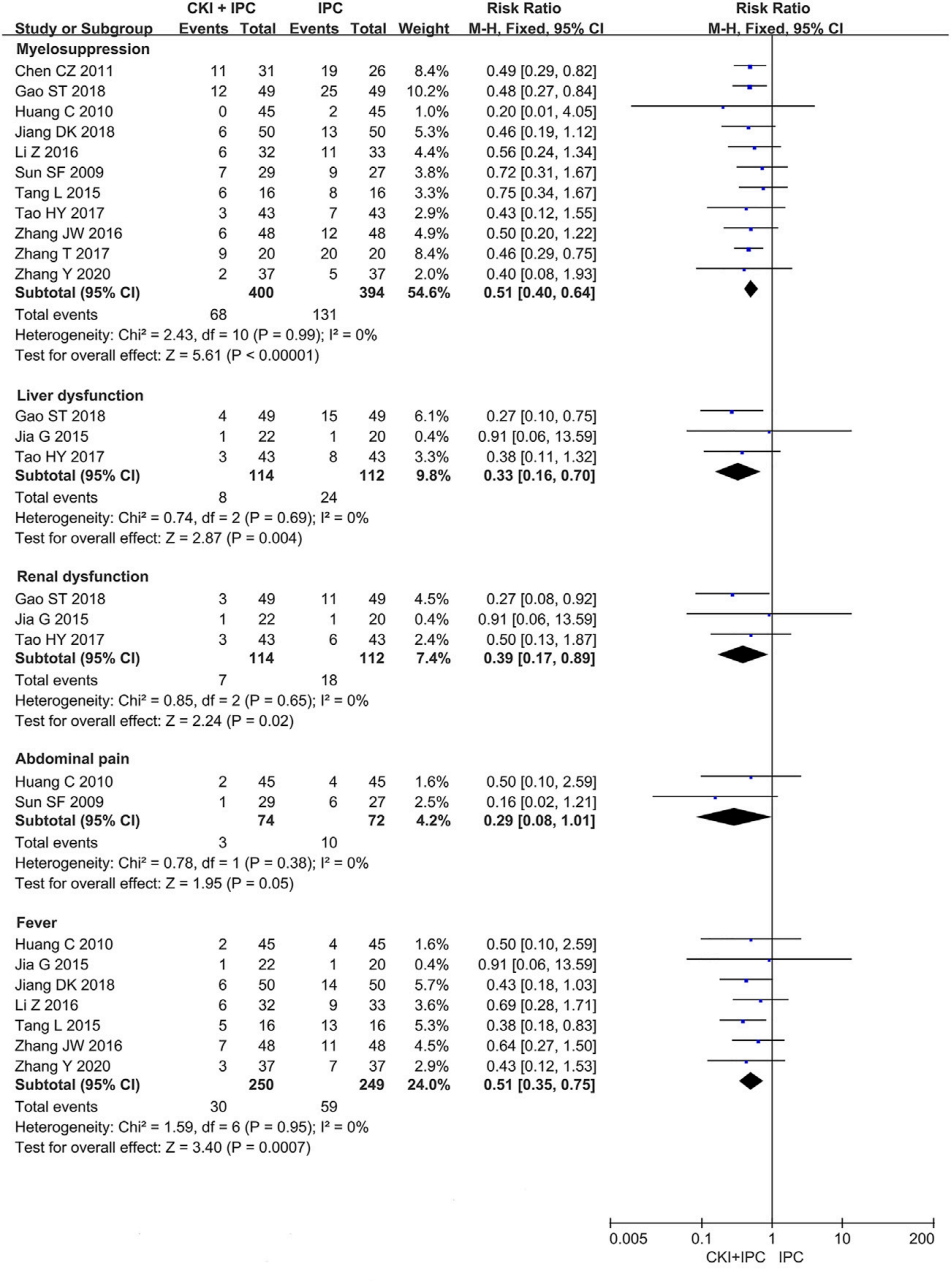
Forest plot and pooled risk ratios for the association of ADRs with CKI + IPC and IPC.

Meta-analysis showed that the incidence of myelosuppression was lower than that in the control group (FEM, RR = 0.51, 95%CI 0.40 to 0.64, 794 participants, *I* ^*2*^ = 0, *p* < 0.00001). The treatment group had an advantage in decreasing the incidence of liver dysfunction (FEM, RR = 0.33, 95%CI 0.16 to 0.70, 226 participants, *I* ^*2*^ = 0, *p* = 0.004), renal dysfunction (FEM, RR = 0.39, 95%CI 0.17 to 0.89, 226 participants, *I* ^*2*^ = 0, *p* = 0.02) and fever (FEM, RR = 0.51, 95%CI 0.35 to 0.75, 499 participants, *I* ^*2*^ = 0, *p* = 0.0007). However, no significant difference was found in the incidence of abdominal pain between groups (FEM, RR = 0.29, 95%CI 0.08 to 1.01, 146 participants, *I* ^*2*^ = 0, *p* = 0.05) (Shown in Figure 7; Table 3).

**TABLE 3:** Meta-analysis results of adverse events.

Outcomes	Number of trials	Experimental group (Events/Total)	Control group (Events/Total)	SM	RR, 95%CI	Z	*P*	Heterogeneity	
								*I* ^*2*^ (%)	*P*_*h*_
Myelosuppression	11	68/400	131/394	FEM	0.51 [0.40,0.64]	5.61	<0.00001	0	0.99
Liver dysfunction	3	8/114	24/112	FEM	0.33 [0.16,0.70]	2.87	0.004	0	0.69
Renal dysfunction	3	7/114	18/112	FEM	0.39 [0.17,0.89]	2.24	0.02	0	0.65
Abdominal pain	2	3/74	10/72	FEM	0.29 [0.08,1.01]	1.95	0.05	0	0.38
Fever	7	30/250	59/249	FEM	0.51 [0.35,0.75]	3.40	0.0007	0	0.95

Note: RR, risk ratio; CI, confidence interval; SM, statistical method; FEM, fixed effects model.

**FIGURE 8:**
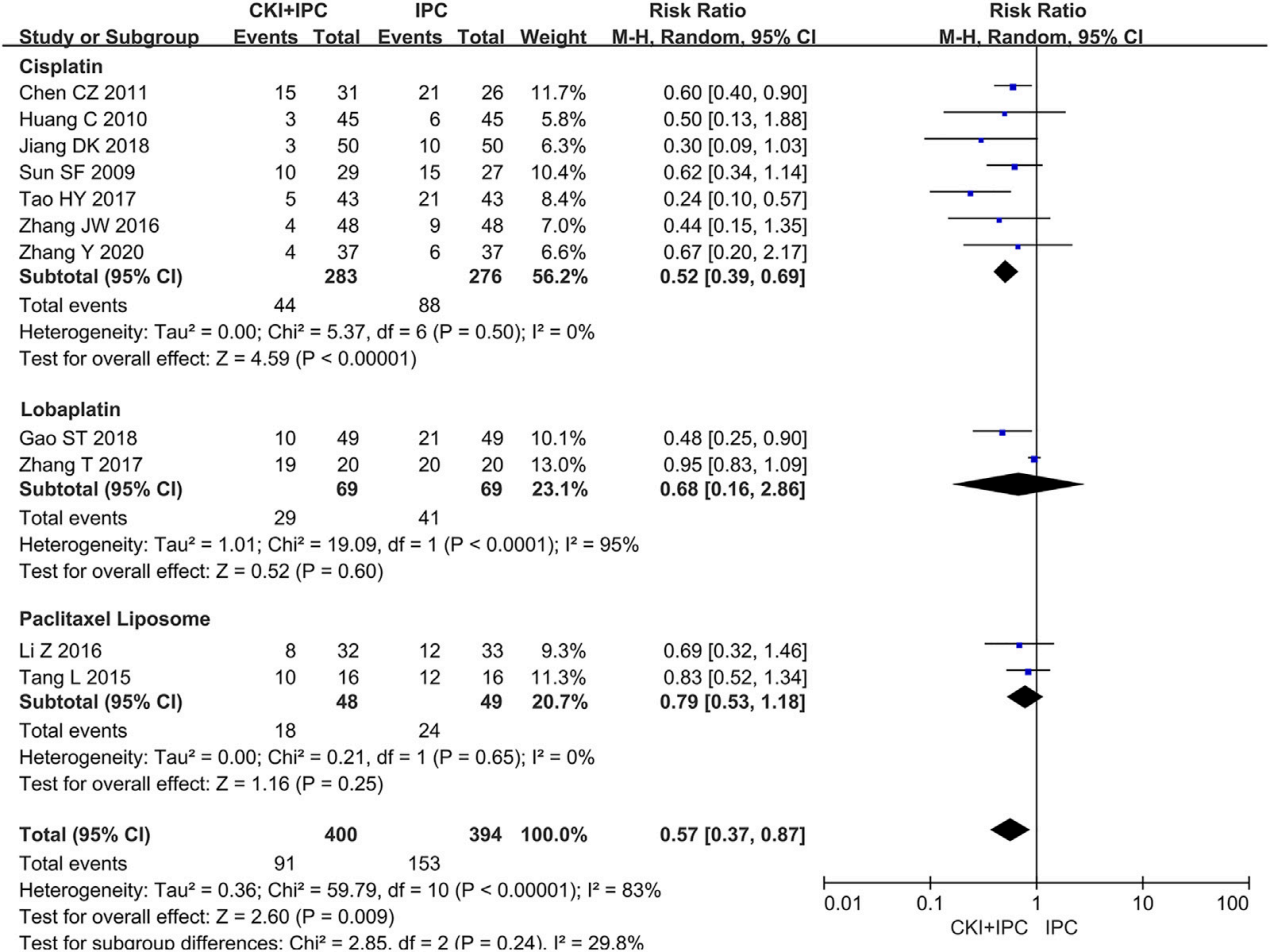
Forest plot and pooled risk ratios for the association of gastrointestinal reactions with CKI + IPC and IPC. Subgroup analysis of different chemotherapy regimen.

Moreover, 794 patients in 11 studies (Sun and Yang, 2009; Huang, 2010; Chen and Chen, 2011; Tang et al., 2015; Li et al., 2016; Zhang et al., 2016; Tao et al., 2017; Zhang D, et al., 2017; Gao S. T et al., 2018; Jiang et al., 2018; Zhang Y et al., 2020) reported gastrointestinal reactions. Owing to the large heterogeneity, we could not conduct a meta-analysis. However, subgroup analysis showed that, when compared with cisplatin alone, CKI combined with cisplatin offered an advantage in decreasing the incidence of gastrointestinal reactions (REM, RR = 0.52, 95%CI 0.39 to 0.69, 559 participants, *I* ^*2*^ = 0%, *p* < 0.00001) (Shown in Figure 8).

### 3.6 Publication bias

**FIGURE 9:**
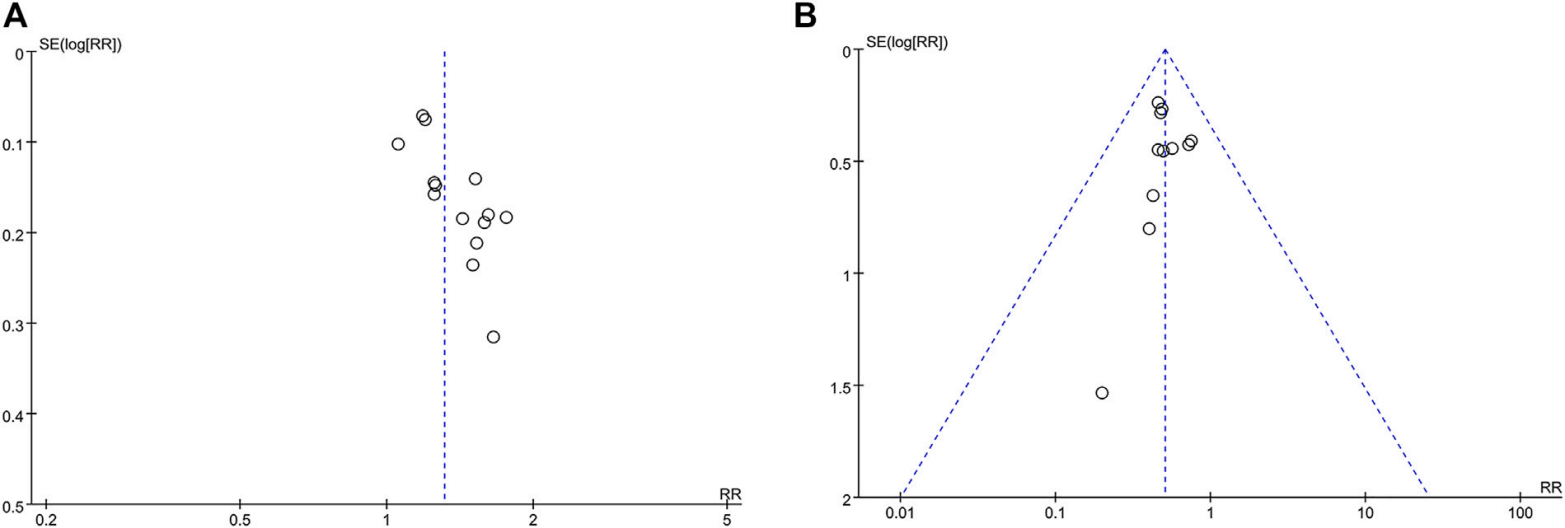
Funnel plot **(A)** CKI + IPC compared to IPC with the ORR reported in 14 trails; **(B)** CKI + IPC compared to IPC with the myelosuppression reported in 11 trails.

When compared with the funnel plot of IPC alone, the ORR and myelosuppression in the studies with CKI combined with IPC were distributed on both the sides symmetrically (Shown in Figure 9). Begg’s test indicated no significant publication bias in the meta-analysis of ORR (*p* = 0.1005) and myelosuppression (*p* = 0.2129).

### 3.7 Sensitivity analyses

Base on the protocol, sensitivity analysis was performed to determine the effect of removing the high-risk study and switching REM/FEM on the results. Only one high risk trial (Chen and Chen, 2011) was included in our review, and the results showed that there was no difference after removing the high-risk trials from the meta-analysis (the result from original Figure 3 to RR = 1.32, 95%CI 1.20 to 1.46, *p* < 0.00001). After switching the REM/FEM, we found that our results were stable.

### 3.8 Quality of evidence

As shown in Table 4, based on the GRADE criteria, the quality of evidence was found to be moderate for ORR, myelosuppression, fever, and low for renal dysfunction, liver dysfunction, and very low for QoL and abdominal pain.

**TABLE 4:** GRADE evidence profile of clinical efficacy and safety.

Outcomes (trials)	Quality assessment					No. of patients		Effect		Quality of evidence
	Risk of bias	Inconsistency	Indirectness	Imprecision	Publication bias	CKI plus IPC	IPC alone	Risk ratios (95% CI)	Difference	
ORR (14)	Serious^a^	NO	NO	NO	NO	424/509 (80.8%)	304/493 (61.7%)	1.31 (1.20–1.43)	19.1% more (12.3 more to 26.5 more)	⊕⊕⊕○
										Moderate
QoL (3)	Serious^a^	Serious^b^	NO	Serious^c^	NO	60/117 (51.3%)	90/117 (76.9%)	1.50 (1.23–1.83)	25.6% more (11.8 more to 42.6 more)	⊕○○○
										Very low
Myelosuppression (11)	Serious^a^	NO	NO	NO	NO	131/394 (33.2%)	68/400 (17.0%)	0.51 (0.40–0.64)	16.3% fewer (19.9 fewer to 12 fewer)	⊕⊕⊕○
										Moderate
Liver damage (3)	Serious^a^	NO	NO	Serious^b^	NO	24/112 (21.4%)	8/114 (7.0%)	0.33 (0.16–0.70)	14.4% fewer (18 fewer to 6.4 fewer)	⊕⊕○○
										Low
Renal damage (3)	Serious^a^	NO	NO	Serious^b^	NO	18/112 (16.1%)	7/114 (6.1%)	0.39 (0.17–0.89)	9.8% fewer (13.3 fewer to 1.8 fewer)	⊕⊕○○
										Low^a^
Abdominal pain (2)	Serious^a^	Serious^d^	NO	Very serious^c^ ^,^ ^e^	NO	10/72 (13.9%)	3/74 (4.1%)	0.29 (0.08–1.01)	9.9% fewer (12.8 fewer to 0.1 more)	⊕○○○
										Very low
Fever (7)	Serious^a^	NO	NO	NO	NO	59/249 (23.7%)	30/250 (12.0%)	0.51 (0.35–0.75)	11.6% fewer (15.4 fewer to 5.9 fewer)	⊕⊕⊕○
										Moderate

^a^ There were serious limitations of methodological quality among trials according to the risk of bias assessment.

^b^ There was significant difference among trials according to effect value.

^c^ Too small simple size.

^d^ There was no difference between the experience group and control group according to *p*-value.

^e^ Too wide confidence interval.

## 4 Discussion

CKI combined with IPC has been widely used for treating patients with MA. However, because of the small sample size and insufficient quality of the single study, the efficacy and safety of this treatment plan should be investigated. Therefore, we conducted this systematic review and meta-analysis after systematic searching and screening and included 17 RCTs. The outcomes included ORR, survival time, immune function, QoL, and ADRs.

CKI is a botanical drug with broad-spectrum and multi-target anti-tumor effect. The main compounds of CKI are matrine (MT) and oxymatrine, which exhibit diverse anti-tumor pharmacological activities (Chen et al., 2022). Basic research showed that CKI could induce tumor apoptosis. Wu, X (Wu et al., 2022). reported that CKI combined with cisplatin exerted a synergistic anti-tumor effect against the p53-R273H/P309S mutant (SW480 cell) in colorectal cancer cells by inducing apoptosis. Yang, Y (Yang et al., 2020). indicated that CKI relieved the immunosuppression of tumor-associated macrophages and promoted the proliferation and cytotoxic ability of CD8^+^ T cells, thus resulting in the apoptosis of hepatocellular carcinoma cells. In addition, CKI could inhibit metastasis and angiogenesis in tumor. Through live cell imaging, Nourmohammadi (Nourmohammadi et al., 2019) confirmed that CKI strongly reduced the migration of HT-29 and MDA-MB-231 cells, moderately slowed brain cancer cells, and had a small effect on HEK-293 cells (Wang Q. G et al., 2021). indicated that CKI intervened in the metabolic reprogramming and epithelial-mesenchymal transition of HCC by regulating *β*-catenin/c-Myc signaling pathway and reducing their migration. CKI can also reduce the angiogenesis in the tumor tissues and play a role in inhibiting tumor growth (Wang et al., 2019). With the increasing CKI dose, the microvessel density of transplanted tumors decreased significantly, whereas the vascular maturity index increased significantly. CKI also regulated the immune functions. By performing an enzyme-linked immunosorbent assay, Shen (Shen et al., 2019) found that CKI could upregulate IL-1β gene, thereby increasing the IL-1β levels. The pathophysiology of MA is a combination of obstructed lymphatic drainage and altered vascular permeability. [12] Base on the present experimental evidence, CKI could inhibit tumor cells to relieve lymphatic obstruction and inhibit neoangiogenesis in the tumor tissues, which could be the mechanism of CKI for controlling MA. Animal experiments have demonstrated the positive effect of CKI on ascites in mice (Zhang L. Q, et al., 2012; Wang K. X et al., 2021). Several clinical trials have also shown that CKI combined with chemotherapy could increase the anti-tumor efficacy, improve QoL, and decrease ADRs (Pu et al., 2019; Zhang D et al., 2020; Zhang et al., 2021), which are consistent with our study results.

Moderate evidence obtained in the meta-analysis showed that CKI combined with IPC was more effective than IPC alone regarding ORR (19.1% more than IPC). The subgroup analysis showed that CKI may exert a better effect when combined with lobaplatin regimen, CKI 40 mL. It also showed that CKI played a better role in treating MA, which may be closely related to the multi-target anti-tumor activities of CKI, as confirmed by some network pharmacology studies. The anti-gastric cancer effect of CKI and its key targets were verified through *in vivo* and *in vitro* experiments. CKI could regulate phosphoinositide-3-kinase (PI3K)/Akt and toll-like receptor signaling pathways by interfering with hub genes such as *AKR1B1*, *MMP2* and *PTGERR3* (Zhou et al., 2021). CKI could exert anti-HCC effects through key targets such as MMP2, MYC, CASP3, REG1A, and the key pathways of glycometabolism and amino acid metabolism (Gao L et al., 2018). Wu, C (Wu et al., 2021). reported that, in PC treatment, the mechanism was related to cell cycle, Janus kinase/signal transducers, and the activators of transcription, ErbB, PI3K/Akt and mammalian target of the rapamycin signaling pathways. Furthermore, CDK1, JAK1, EGFR, MAPK1 and MAPK3 served as the core genes regulated by CKI in PC treatment.

In this review, we included “survival time” as an outcome, which is different from the protocol, for the following reasons: First, in a later study, we found that most patients with MA had a shorter survival time (approximately 5.7 months). (Hodge and Badgwell, 2019). We propose the inclusion of “survival time” as one of the outcomes to obtain evidence on the effect of CKI combined with IPC on this outcome in patients with MA. Second, “survival time” is one of the recommended evaluation measures for the clinical trials of cancer-related drugs according to the U.S. Food and Drug Administration (Services et al., 2018) and other studies (Wilson, et al., 2015a; Wilson, et al., 2015). “Survival time” is an important outcome for evaluating drug efficacy in many cancers. After comprehensive consideration, we decided to include this outcome in the scope of the review and truthfully reported the results.

None of the studies included in this review reported survival times. Most of them consisted of treatments that lasted between 1 and 2 months. However, the median survival of patients with MA was 5.7 months (Hodge and Badgwell, 2019). Several researchers have not yet observed this outcome and have neglected to follow up these patients. Nevertheless, several studies have demonstrated the positive effect of CKI on prolonging the survival of patients with multiple cancers. The meta-analysis revealed that CKI combined with platinum-based chemotherapy could improve the 1-year survival rate of patients with advanced non-small cell lung cancer (RR = 1.51, 95% CI 1.18 to 1.94, *p* = 0.001) (Chen et al., 2020). Some clinical studies have shown that CKI combined with chemotherapy could prolong the survival of patients with MA (Chen et al., 2011; Zhang et al., 2015). Furthermore, past studies have shown that CKI combined with chemotherapy could prolong the survival of patients with non-small cell lung cancer (Xiao et al., 2012), cervical cancer (Yang et al., 2018), and bladder cancer (Liu, 2018). Zhang Y found that CKI may improve the survival of Ehrlich ascites carcinoma in mice by exerting an antioxidant effect (Zhang W. J, et al., 2012). These clinical and elementary studies indicate that CKI might potentially prolong the survival of patients with MA, albeit this warrants further exploration.

With the popularization of bio-psycho-social medicine model, doctors, and patients are paying attention to the QoL, which is considered an additional outcome to evaluate cancer treatment by several researchers (Anota et al., 2015). MA is a poor prognostic indicator and detrimental to the QoL (Hodge and Badgwell, 2019). In this meta-analysis, very low evidence indicated that CKI combined with IPC might improve the QoL of patients with MA (25.6% more than IPC). The subgroup analysis showed that the effect of the 30 mL subgroup of CKI was stronger than that of the 40 mL subgroup of CKI. We found only one study in the 40 mL dose subgroup, and the present results are based on the differences between the two groups after treatment. However, as the baseline KPS score of the 40 mL subgroup was lower than that of the 30 mL subgroup in the original study, the general condition of the patients was worse, and the degree of improvement was poor, which may explain the lower effect of the 40 mL subgroup. We found that the dose of CKI may be a factor affecting the QoL of patients with MA; however, we could not conclude the specific extent of the effect based on the present data. The results regarding the effect of CKI dose on QoL improvement warrants further investigation.

Due to the limitations of the original study, although some clinical (Zhao et al., 2016) and animal studies (Zhou et al., 2012) have suggested that CKI may enhance immune function, we cannot conclude the effect of CKI combined with IPC on immune functions. In this study, one study showed different results from the other three in terms of the characteristics studied based on the level of CD8^+^, implying the uncertainty of the effect of CKI combined with IPC on immune function. Thus, the effect of CKI on immune functions warrants further exploration.

The symptoms of MA are generally caused by abdominal distention, visceral compression, and the loss of proteins and electrolytes. These symptoms include abdominal pain, nausea, vomiting, dyspnea, anorexia, dyskinesia, and fatigue, which severely affects the QoL of patients with MA (Cavazzoni et al., 2013). The decrease in the QoL of patients with MA can be alleviated by treating ascites itself (Hodge and Badgwell, 2019). Interestingly, the effect of CKI on QoL may also be related to energy metabolism. Previous studies have demonstrated that CKI could affect energy synthesis by regulating glucose metabolism, which may be the mechanism for improving the QoL. Cui, J (Cui et al., 2019). found reduced energy metabolism in cancer cells based on reduced glucose consumption and cellular energy charges. CKI might improve the QoL by increasing the immune function of patients with cancer. In an experiment, Shen (Zhou et al., 2012) indicated that CKI could blocks gastric carcinogenesis, thereby protecting against carcinogen-induced oxidative damage and improving immunity.

IPC is a widely used and effective treatment strategy for MA. However, not all patients can benefit from it because of intolerant ADRs to chemotherapy drugs, including gastrointestinal reactions, myelosuppression, liver and renal dysfunction, abdominal pain, and fever. Moreover, the expected treatment cycle could not be achieved because of severe ADRs, which may confuse physicians. In this study, moderate evidence supports that the combination group exhibited a lower incidence of myelosuppression (16.3% lower than the control group) and fever (11.6% lower than the control group) in patients with MA. Due to the huge heterogeneity, we could not conduct a meta-analysis for gastrointestinal reactions. However, the subgroup analysis showed that CKI combined with cisplatin could decrease the incidence of gastrointestinal reactions when compared with cisplatin alone (RR = 0.52, *p* < 0.00001). In addition, less evidence indicated that the combination group showed a lower incidence of liver dysfunction (14.4% lower than the control group) and renal dysfunction (9.8% lower than the control group), but it did not show an increased burden of abdominal pain (RR = 0.29, *p* = 0.05). Saeed Nourmohammadi (Nourmohammadi et al., 2019) reported that CKI uniformly blocked invasiveness *via* the extracellular matrix. CKI increased apoptosis in breast cancer cells, but not in the non-cancerous cell lines. CKI did not affect the viability of all cell lines, which may explain why CKI did not increase the burden of ADRs. To summarize, our cumulative results indicate that CKI combined with IPC is safe and can reduce the incidence of some ADRs in MA treatment.

Although several systematic reviews published previously are similar to our research topics, they are all different from this review. Methodologically, we used a recognized authoritative tool (GRADE criteria) and performed subgroup analysis and meta-regression to further explore the sources of heterogeneity. The present study conforms to the PRISMA guideline (McInnes et al., 2018). Considering the evidence obtained, our study is newer and more standardized than related past studies. Moreover, unlike previous studies, we included additional outcomes such as the survival time and immune functions in the evaluation. For evaluating the safety of CKI, we included more types of adverse events than the previous studies did. Moreover, Wang Peipei (Wang, 2020) focused on the differences between the efficacies of different botanical injections, whereas we focused on the efficacy and safety of CKI combined with IPC. Tang Ziwei (Tang, 2020) only evaluated CKI combined with cisplatin for treating MA. Xu Zhong (Xu et al., 2014) included some studies in which intravenous CKIs were used. Some studies reported that the different routes of drug administration may affect drug efficacy and safety outcomes (Dai et al., 2020). Therefore, to reduce bias, our inclusion criteria were the intraperitoneal infusion of both CKI and chemotherapy drugs. Moreover, previous studies have indicated the exact efficacy of CKI combined with chemotherapy regimen for treating liver cancer (Ma et al., 2016), digestive tract tumors (Zhang et al., 2021), breast cancer (Liu et al., 2020), lung cancer (Li et al., 2022), gastric cancer (Zhang T et al., 2017), and cancer pain (Yanju et al., 2014), which indirectly supports the results. We hope to provide the best and latest available evidence by adopting evidence-based medicine methods and providing clinicians with a clear and effective treatment plan.

We have some instructions for future research. First, the present study has some limitations regarding the methodological quality of the trials for the risk of bias assessment. Most studies possessed an unclear risk regarding factors including random sequence generation, allocation concealment, blinding of participants and personnel, and blinding of the outcome assessment. Future studies should focus on applying random methods, implementing blinding methods, and properly managing missing data (e.g., intention to treat). Reporting baseline data (e.g., sex, age, type of tumor, histological type, neoplasm staging of patients) and drug manufacturer information are also essential. Registration of clinical trials is also necessary so that more information about the study design can be made public. Furthermore, researchers should conduct well-designed and high-quality clinical studies per the CONSORT guidelines (Schulz et al., 2010) to verify the efficacy and safety of CKI combined with IPC for treating MA. Second, MA is the manifestation of tumor deterioration, and the survival time of such patients is usually short. Thus, the survival data of these patients should be observed and reported. Third, most of the studies included in this review only reported ADR incidence. We recommend reporting ADRs using standardized scales and/or criteria such as the National Cancer Institute-Common Toxicity Criteria for Adverse Events to better reflect the extent of ADRs. Fourth, we recommend that patients with the same type of cancer should be considered as a study population and those with different types of cancers should be reported by subgroups to decrease the effect of cancer types on outcomes including the survival time and ORR. Finally, as we found that the dose of CKI may affect the QoL of patients, we suggest that subsequent analyses should further clarify the relationship between the dose of CKI and QoL.

The study has the following limitations. First, we have only searched English and Chinese databases. All included studies are from China, which may have incurred regional and ethnic differences. Second, most studies included in this review may not strictly adhere to the CONSORT reporting standards; hence, some results are rated as low or very low at the time of GRADE rating. Third, this study was not a reticular meta-analysis; hence, we could not evaluate the difference between the efficacies of CKI and other botanical injections. However, the cluster analysis of 29 RCTs (Zhang D et al., 2020) of 8 botanical injections showed that CKI combined with chemotherapy was the optimal choice for improving the clinical efficacy rate and ADRs in patients with esophageal cancer. Finally, most studies included MA caused by multiple types of tumors as a study population, which may have increased the confounding bias. Hence, subsequent studies should address these limitations.

## 5 Conclusion

This study result suggests that CKI combined with IPC can increase ORR and improve QoL of patients with MA. In addition, this combination treatment can partially reduce toxicity caused by chemotherapy drugs. However, the efficacy and safety of CKI combined with IPC for patients with MA needs to be verified in future by conducting well-designed and high-quality clinical trials that adhere to the CONSORT guidelines.

## Data Availability

The original contributions presented in the study are included in the article/Supplementary Material, further inquiries can be directed to the corresponding author.

## Abbreviations

CKI, Compound Kushen Injection; IPC, intraperitoneal chemotherapy; MA, malignant ascites; RCTs, randomized controlled trials; ORR, objective response rate; QoL, quality of life; ADRs, adverse drug reactions; GRADE, Grading of Recommendations Assessment Development and Evaluation criteria; CI, confidence intervals; RR, risk ratio; FDA, Food and Drug Administration; CFDA, China Food and Drug Administration; CR, complete response; PR, partial response; SD, stable disease; PD, progressive disease; MD, mean difference; KPS, Karnofsky Performance Status; FEM, fixed-effects model; REM, random-effects model; MT, matrine; OMT, oxymatrine; CRC, colorectal cancer cells; MVD, micro vessel density; PC, pancreatic cancer; OS, overall survival; PFS, progression-free survival; DFS, Disease-free survival; HCC, Hepatocellular Carcinoma; SM, statistical method.
