# Supplementary material for: Evaluation of efficacy and safety for compound kushen injection combined with intraperitoneal chemotherapy for patients with malignant ascites: A systematic review and meta-analysis
Source: Front Pharmacol. 2023 Mar 3;14:1036043. doi: 10.3389/fphar.2023.1036043 (PMC10020185; doi:10.3389/fphar.2023.1036043)
Supplement: Supplementary file 2 [file Table2.DOCX]

**Supplementary material 2. Detailed search strategy**

**Supplementary Table A: Search Strategy Used in PubMed 2023/1/20**

| No. | Search items | Items found |
| --- | --- | --- |
| #1 | "ascitic fluid"[Mesh] OR "ascites"[Mesh] OR "ascitic fluid*" [Title/Abstract] OR "peritoneal fluid*" [Title/Abstract] OR "peritoneal effusion*" [Title/Abstract] | 35608 |
| #2 | "matrine"[Supplementary Concept] OR "sophora"[MeSH Terms] OR "kushen"[Title/Abstract] OR "yanshu"[Title/Abstract] OR "matrine"[Title/Abstract] OR "CKI"[Title/Abstract] OR "sophora flavescens"[Title/Abstract] OR "sophorae"[Title/Abstract] OR "sophoridine"[Title/Abstract] | 2970 |
| #3 | ("randomized controlled trial"[Publication Type] OR "controlled clinical trial"[Publication Type] OR "randomized"[Title/Abstract] OR "placebo"[Title/Abstract] OR "drug therapy"[MeSH Terms] OR "randomly"[Title/Abstract] OR "trial"[Title/Abstract] OR "groups"[Title/Abstract]) NOT ("animals"[MeSH Terms] NOT "humans"[MeSH Terms]) | 4049005 |
| #4 | #1 and #2 and #3 | 0 |

**Supplementary Table B. Search Strategy Used in EMBASE 2023/1/20**

| **No.** | **Search items** | **Items found** |
| --- | --- | --- |
| #1 | 'ascites fluid'/exp | 10220 |
| #2 | 'ascites'/exp | 63250 |
| #3 | 'ascitic fluid*' OR 'peritoneal fluid*' OR 'peritoneal effusion*' | 18209 |
| #4 | #1 OR #2 OR #3 | 82075 |
| #5 | 'matrine'/exp | 1620 |
| #6 | 'sophora'/exp | 1323 |
| #7 | kushen:ti,ab,kw OR yanshu:ti,ab,kw OR matrine:ti,ab,kw OR cki:ti,ab,kw OR 'sophora flavescens':ti,ab,kw OR sophorae:ti,ab,kw | 3366 |
| #8 | #5 OR #6 OR #7 | 4406 |
| #9 | random* | 2140104 |
| #10 | #4 AND #8 AND #9 | 4 |

**Supplementary Table C. Search Strategy Used in** **Cochrane 2023/1/20**

| **No.** | **Search items** | **Items found** |
| --- | --- | --- |
| **Search Terms to Pancreatic Cancer:** | | |
| #1 | MeSH descriptor: [ascites] explode all trees | 464 |
| #2 | MeSH descriptor: [ascitic fluid] explode all trees | 116 |
| #3 | ("ascitic fluid*"):ti,ab,kw | 295 |
| #4 | ("peritoneal fluid*"):ti,ab,kw | 938 |
| #5 | ("peritoneal effusion*"):ti,ab,kw | 139 |
| #6 | #1 OR #2 OR #3 OR #4 OR #5 | 1644 |
| #7 | MeSH descriptor: [sophora] explode all trees | 16 |
| #8 | (kushen):ti,ab,kw | 49 |
| #9 | (yanshu):ti,ab,kw | 19 |
| #10 | (matrine):ti,ab,kw | 45 |
| #11 | (CKI):ti,ab,kw | 21 |
| #12 | ("sophora flavescens"):ti,ab,kw | 25 |
| #13 | (sophorae):ti,ab,kw | 28 |
| #14 | (sophoridine):ti,ab,kw | 0 |
| #15 | #7 OR #8 OR #9 OR #10 OR #11 OR #12 OR #13 OR #14 | 177 |
| #16 | #6 AND #15 | 0 |

**Supplementary Table D: Search Strategy Used in CBM 2023/1/20**

**Items found 55**

("随机"[全部字段:智能] OR "RCT"[全部字段:智能]) AND ("腹水"[全部字段:智能] OR "腹腔积液"[全部字段:智能]) AND ("苦参"[全部字段:智能] OR "岩舒"[全部字段:智能])

**Supplementary E: Search Strategy Used in CNKI 2023/1/20**

**Items found 612**

(SU = '苦参' OR SU = '岩舒' OR TKA = '苦参' OR TKA = '岩舒') AND (SU = '腹水' OR SU = '腹腔积液' OR FT = '腹水' OR FT = '腹腔积液') AND ( FT = '随机' OR FT = 'RCT')

**Supplementary Table F: Search Strategy Used in WangFang 2023/1/20**

**Items found 138**

(主题:(苦参) or 主题:(岩舒) or 摘要:(苦参) or 摘要:(岩舒)) and (主题:(腹水) or 主题:(腹腔积液) or 全部:(腹水) or 全部:(腹腔积液))

**Supplementary G: Search Strategy Used in VIP 2023/1/20**

**Items found 112**

(R="苦参" OR R="岩舒") AND (U=腹水 OR U=腹腔积液)

**Supplementary Table H. Search Strategy Used in Clinicaltrials 2023/1/20**

**Items found 0**

Condition or disease: yanshu OR matrine OR sophora OR kushen OR CKI OR sophora flavescens OR sophorae OR sophoridine |Other terms: ascitic fluid OR ascites OR peritoneal fluid OR peritoneal effusion

**Supplementary I: Search Strategy Used in Chinese Clinical Trial Registry 2023/1/20**

**Items found 0**

**注册题目：苦参 + 腹水**

**Supplementary J: Search Strategy Used in** **TRIP medical database 2023/1/20**

**Items found 3**

("ascitic fluid" OR "ascites" OR "ascitic fluid*" OR "peritoneal fluid*" OR "peritoneal effusion*") AND ("matrine" OR "sophora" OR "kushen" OR "yanshu" OR "matrine" OR "CKI" OR "sophora flavescens" OR "sophorae" OR "sophoridine")

**Supplementary K: Search Strategy Used in Latin American and Caribbean Health Sciences Literature (LILACS) 2023/1/20**

**Items found 0**

("ascitic fluid" OR "ascites" OR "ascitic fluid*" OR "peritoneal fluid*" OR "peritoneal effusion*") AND ("matrine" OR "sophora" OR "kushen" OR "yanshu" OR "matrine" OR "CKI" OR "sophora flavescens" OR "sophorae" OR "sophoridine")

**Supplementary L: Search Strategy Used in** **Alt HealthWatch 2023/1/20**

**Items found 12**

("ascitic fluid" OR "ascites" OR "ascitic fluid*" OR "peritoneal fluid*" OR "peritoneal effusion*") AND ("matrine" OR "sophora" OR "kushen" OR "yanshu" OR "matrine" OR "CKI" OR "sophora flavescens" OR "sophorae" OR "sophoridine")

**Supplementary L: Search Strategy Used in Web of Science 2023/1/20**

| No. | Search items | Items found |
| --- | --- | --- |
| #1 | TS=("ascites" OR "ascitic fluid*" OR "peritoneal fluid*" OR "peritoneal effusion*") | 50513 |
| #2 | TS=("matrine" OR "sophora" OR "kushen" OR "yanshu" OR "CKI" OR "sophora flavescens" OR "sophorae" OR "sophoridine") | 4755 |
| #3 | #1 and #2 | 1 |

**Supplementary L: Search Strategy Used in Google Scholar 2023/1/20**

**Items found 3**

("ascitic fluid" OR "ascites" OR "ascitic fluid*" OR "peritoneal fluid*" OR "peritoneal effusion*") AND ("matrine" OR "sophora" OR "kushen" OR "yanshu" OR "matrine" OR "CKI" OR "sophorae" OR "sophoridine") AND ("random")
