# Supplementary material for: Evaluation of efficacy and safety for compound kushen injection combined with intraperitoneal chemotherapy for patients with malignant ascites: A systematic review and meta-analysis
Source: Front Pharmacol. 2023 Mar 3;14:1036043. doi: 10.3389/fphar.2023.1036043 (PMC10020185; doi:10.3389/fphar.2023.1036043)
Supplement: Supplementary file 3 [file Table3.DOCX]

Table S1 Effect of value for immune function of CKI combined with IPC versus IPC alone

| **Study** | **CKI+IPC** | | | **IPC** | | | **Weight** | **Risk ratio** |
| --- | --- | --- | --- | --- | --- | --- | --- | --- |
|  | **Mean** | **SD** | **Total** | **Mean** | **SD** | **Total** |  | **M-H, Random,95% CI** |
| 1. CD3+ |  |  |  |  |  |  |  |  |
| Jiang DK 2018 | 50.24 | 3.67 | 50 | 40.91 | 2.26 | 50 | 0.2% | 9.33 [8.14, 10.52] |
| Zhang JW 2016 | 62.86 | 5.76 | 48 | 59.34 | 5.4 | 48 | 0.1% | 3.52 [1.29, 5.75] |
| Zhang LQ 2012 | 65.63 | 6.27 | 27 | 51.32 | 5.68 | 21 | 0.0% | 14.31 [10.92, 17.70] |
| Zhang Y 2020 | 53.67 | 5.21 | 37 | 48.31 | 5.24 | 37 | 0.1% | 5.36 [2.98, 7.74] |
| 1. CD4+ |  |  |  |  |  |  |  |  |
| Jiang DK 2018 | 37.46 | 3.51 | 50 | 29.9 | 2.08 | 50 | 0.2% | 7.56 [6.43, 8.69] |
| Zhang JW 2016 | 38.82 | 5.41 | 48 | 30.15 | 4.63 | 48 | 0.1% | 8.67 [6.66, 10.68] |
| Zhang LQ 2012 | 58.74 | 5.11 | 27 | 44.34 | 4.53 | 21 | 0.0% | 14.40 [11.67, 17.13] |
| Zhang Y 2020 | 50.71 | 5.01 | 37 | 47.32 | 5.08 | 37 | 0.1% | 3.39 [1.09, 5.69] |
| 3. CD8+ |  |  |  |  |  |  |  |  |
| Jiang DK 2018 | 28.5 | 4.18 | 50 | 34.86 | 5.39 | 50 | 0.1% | -6.36 [-8.25, -4.47] |
| Zhang JW 2016 | 21.62 | 3.11 | 48 | 26.2 | 3.46 | 48 | 0.2% | -4.58 [-5.90, -3.26] |
| Zhang LQ 2012 | 35.27 | 3.87 | 27 | 26.67 | 2.85 | 21 | 0.1% | 8.60 [6.70, 10.50] |
| Zhang Y 2020 | 21.38 | 2.18 | 37 | 23.26 | 2.21 | 37 | 0.3% | -1.88 [-2.88, -0.88] |
| 4. CD4+/CD8+ |  |  |  |  |  |  |  |  |
| Jiang DK 2018 | 1.29 | 0.36 | 50 | 0.84 | 0.24 | 50 | 22.1% | 0.45 [0.33, 0.57] |
| Zhang JW 2016 | 1.79 | 0.19 | 48 | 1.15 | 0.21 | 48 | 49.5% | 0.64 [0.56, 0.72] |
| Zhang LQ 2012 | 1.94 | 0.38 | 27 | 1.65 | 0.42 | 21 | 6.0% | 0.29 [0.06, 0.52] |
| Zhang Y 2020 | 2.06 | 0.29 | 37 | 1.81 | 0.25 | 37 | 20.9% | 0.25 [0.13, 0.37] |
| 5. NK |  |  |  |  |  |  |  |  |
| Zhang JW 2016 | 18.32 | 4.14 | 48 | 15.41 | 4.19 | 48 | 0.1% | 2.91 [1.24, 4.58] |


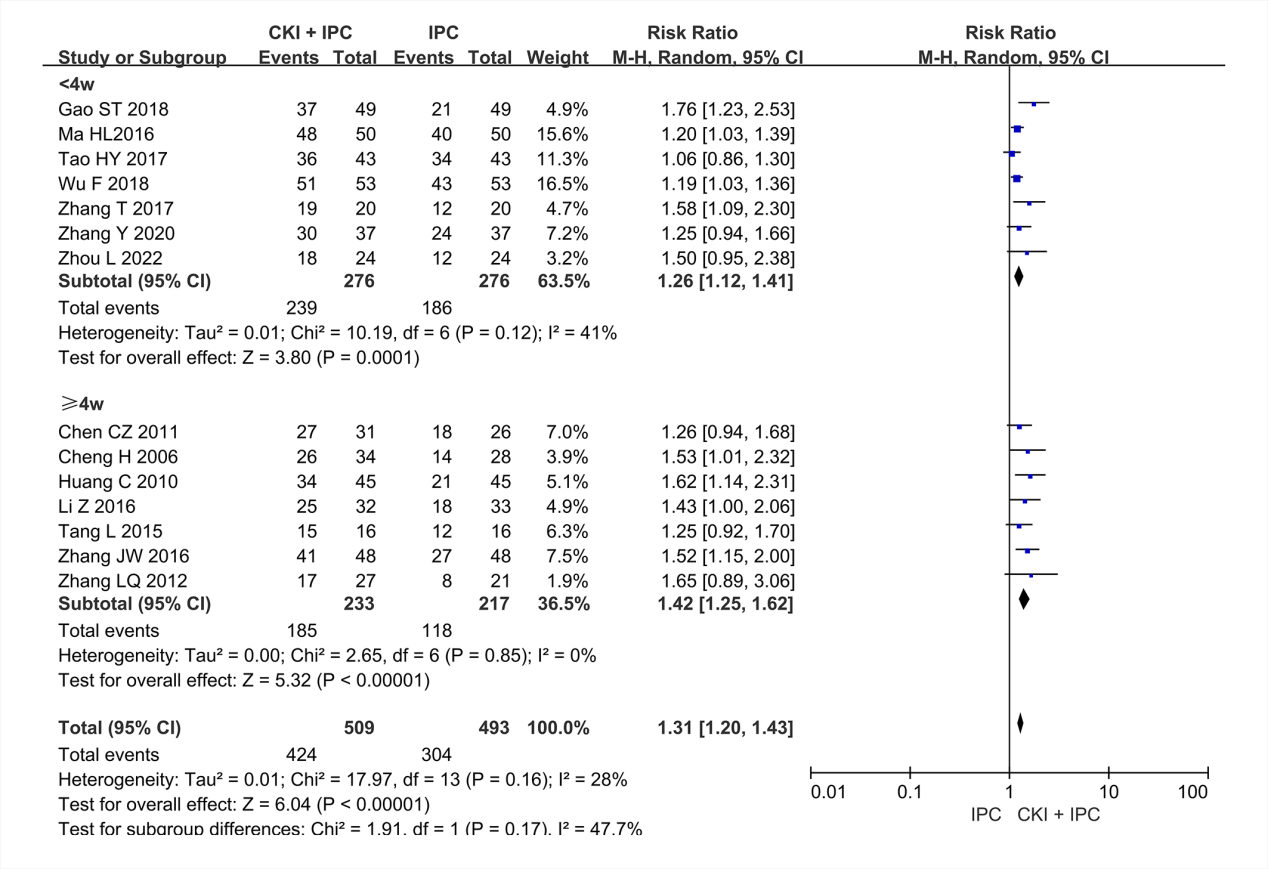


**FIGURE S1 │** Meta-analysis results of ORR between the two groups. Subgroup analysis of different follow-up time.


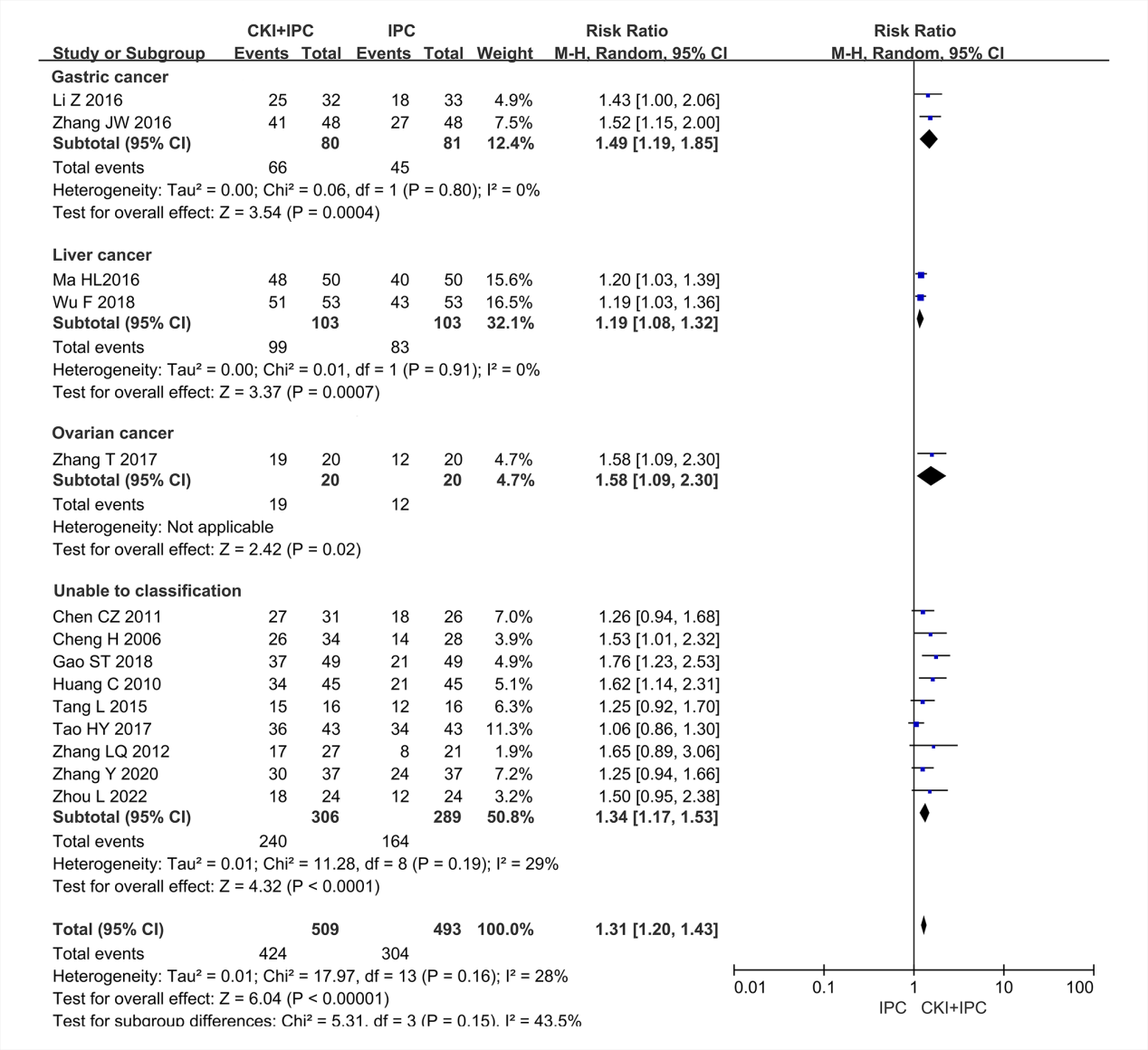


**FIGURE S2 │** Meta-analysis results of ORR between the two groups. Subgroup analysis of different cancer types.


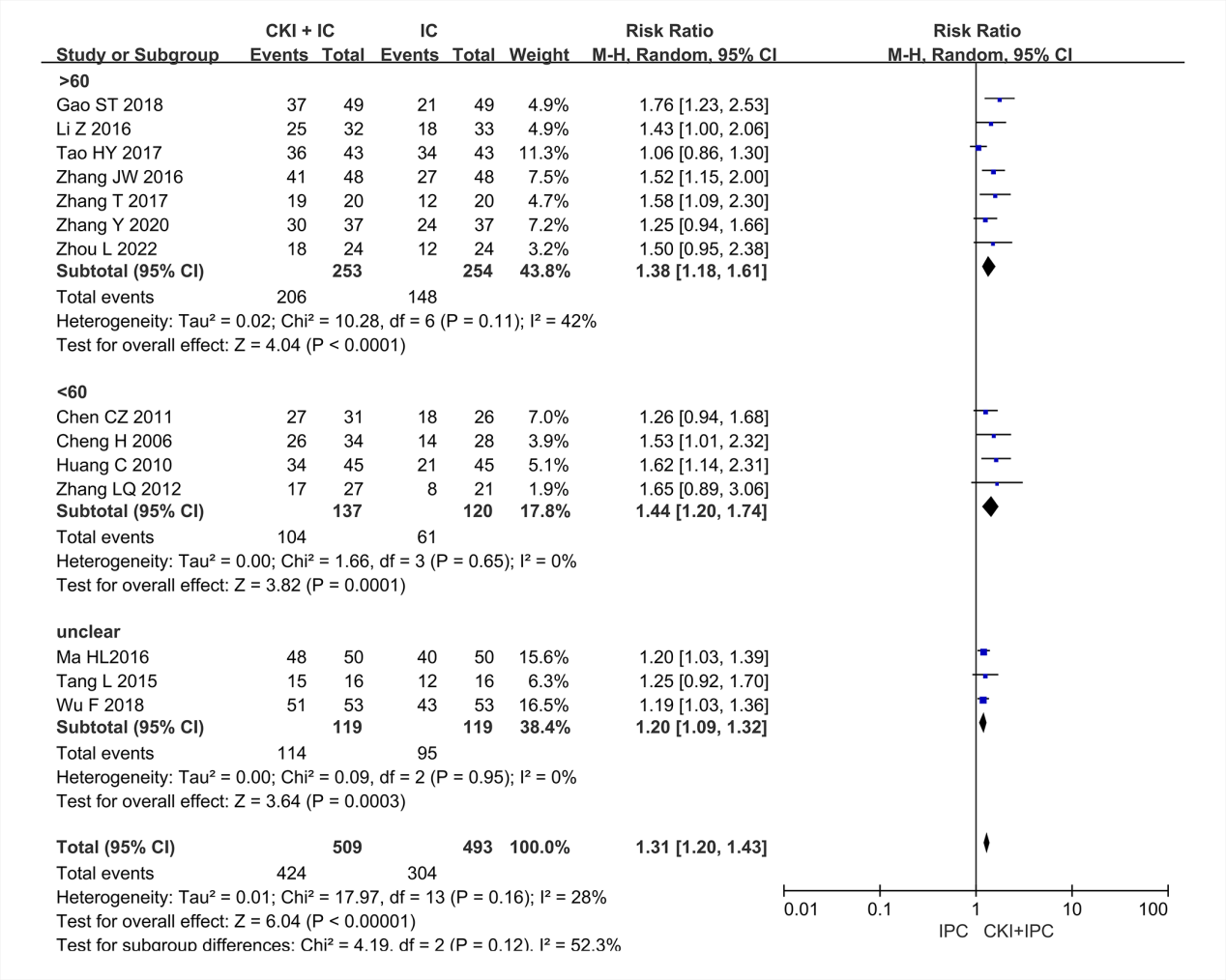


**FIGURE S3 │** Meta-analysis results of ORR between the two groups. Subgroup analysis of different KPS score.


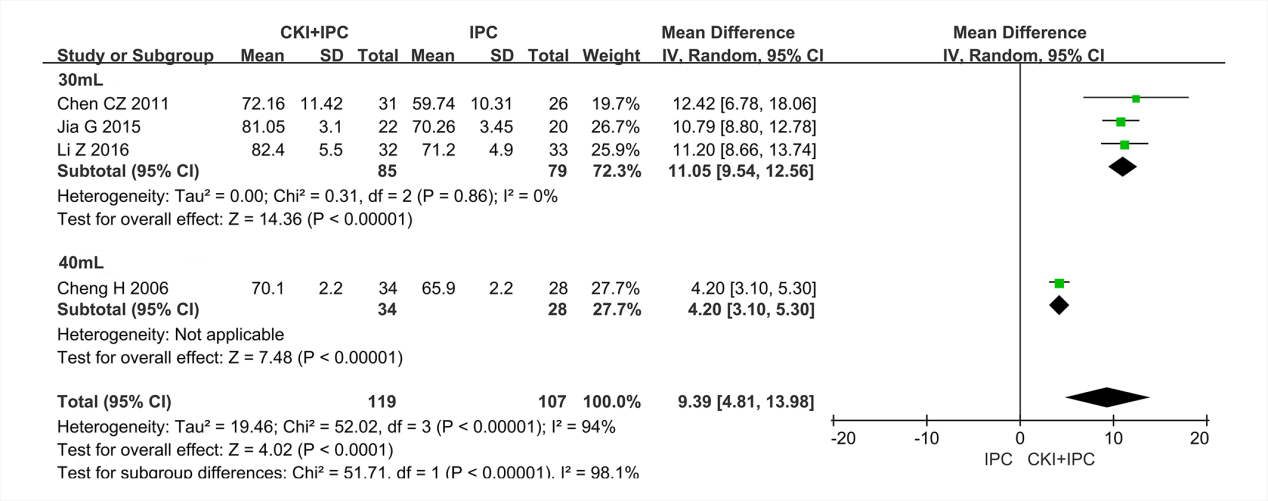


**FIGURE S4 │** Meta-analysis results of QoL(continuous data) between the two groups. Subgroup analysis of different CKI dosage.
